# *Arabidopsis thaliana* Response to Extracellular DNA: Self Versus Nonself Exposure

**DOI:** 10.3390/plants10081744

**Published:** 2021-08-23

**Authors:** Maria Luisa Chiusano, Guido Incerti, Chiara Colantuono, Pasquale Termolino, Emanuela Palomba, Francesco Monticolo, Giovanna Benvenuto, Alessandro Foscari, Alfonso Esposito, Lucia Marti, Giulia de Lorenzo, Isaac Vega-Muñoz, Martin Heil, Fabrizio Carteni, Giuliano Bonanomi, Stefano Mazzoleni

**Affiliations:** 1 Department of Agricultural Sciences, University of Naples Federico II, Via Università 100, 80055 Portici, Italy; f.monticolo90@gmail.com (F.M.); fabrizio.carteni@unina.it (F.C.); giuliano.bonanomi@unina.it (G.B.); 2 Department of Research Infrastructures for Marine Biological Resources (RIMAR), Stazione Zoologica “Anton Dohrn”, 80121 Napoli, Italy; emanuela.palomba@szn.it; 3 Department of Agri-Food, Animal and Environmental Sciences, University of Udine, 33100 Udine, Italy; guido.incerti@uniud.it; 4 Telethon Institute of Genetics and Medicine, via campi Flegrei, 34 Pozzuoli, 80078 Napoli, Italy; chiara.colantuono@gmail.com; 5 Institute of Biosciences and Bioresources (IBBR), National Research Council of Italy (CNR), 80055 Portici, Italy; pasquale.termolino@ibbr.cnr.it; 6 Biology and Evolution of Marine Organisms Department (BEOM), Stazione Zoologica “Anton Dohrn”, 80121 Napoli, Italy; gio.benv@szn.it; 7 Dipartimento di Scienze della Vita, University of Trieste, 34127 Trieste, Italy; alessandro.foscari@phd.units.it; 8 Department of Cellular, Computational and Integrative Biology—CIBIO, University of Trento, 38123 Trento, Italy; alfonso.esposito@unitn.it; 9 Department of Biology and Biotechnology “C. Darwin”, Sapienza University of Rome, 00185 Rome, Italy; lucia.marti@uniroma1.it (L.M.); giulia.delorenzo@uniroma1.it (G.d.L.); 10 Departemento de Ingeniería Genética, CINVESTAV-Irapuato, Guanajuato 36821, Mexico; vega.aphelion@gmail.com (I.V.-M.); mheil@ira.cinvestav.mx (M.H.)

**Keywords:** exDNA, environmental DNA, DNA sensing, self-DNA inhibition, autotoxicity, plant response, DAMP, PAMP, EDAP

## Abstract

The inhibitory effect of extracellular DNA (exDNA) on the growth of conspecific individuals was demonstrated in different kingdoms. In plants, the inhibition has been observed on root growth and seed germination, demonstrating its role in plant–soil negative feedback. Several hypotheses have been proposed to explain the early response to exDNA and the inhibitory effect of conspecific exDNA. We here contribute with a whole-plant transcriptome profiling in the model species *Arabidopsis thaliana* exposed to extracellular self- (conspecific) and nonself- (heterologous) DNA. The results highlight that cells distinguish self- from nonself-DNA. Moreover, confocal microscopy analyses reveal that nonself-DNA enters root tissues and cells, while self-DNA remains outside. Specifically, exposure to self-DNA limits cell permeability, affecting chloroplast functioning and reactive oxygen species (ROS) production, eventually causing cell cycle arrest, consistently with macroscopic observations of root apex necrosis, increased root hair density and leaf chlorosis. In contrast, nonself-DNA enters the cells triggering the activation of a hypersensitive response and evolving into systemic acquired resistance. Complex and different cascades of events emerge from exposure to extracellular self- or nonself-DNA and are discussed in the context of Damage- and Pathogen-Associated Molecular Patterns (DAMP and PAMP, respectively) responses.

## 1. Introduction

Mazzoleni and co-workers [1] reported evidence that fragmented exDNA, accumulating in litter during the decomposition process, produces a concentration dependent, species-specific inhibitory effect, reducing root growth and seed germination of conspecifics. This discovery was also extended to different organisms other than plants, including microbes, fungi, protozoa and insects [2]. Such findings have relevant implications for plant-soil ecological theories, providing a chemical basis for autotoxicity [3] among the mechanisms of plant–soil negative feedback [4], and unexpected new functional roles of exDNA and its sensing at cellular level [5], in species interactions at community [6] and ecosystem [7] levels, with an impact on biomedical and biotechnological applications, and is thus deserving further investigation [8,9,10].

It is well known that exDNA is abundant in many habitats, including soil, sediments, oceans and freshwater [11,12,13]. In soil, it can persist over long periods of time [14] due to its binding to the mineral and humic fractions. The environmental DNA originates from the active release or decomposition and recycling of organic matter produced by the whole range of taxa inhabiting the belowground habitat (bacteria, archaea, fungi, protozoa, soil invertebrates and plants) [15]. The persistent nature of exDNA permits its exploitation for the assessment of microbial community composition, soil and plant biodiversity, as well as taxonomic and phylogenetic studies [11,16,17,18].

The exDNA evolutionary role has been discussed in relation to the well-reported process of horizontal gene transfer among microbial populations [15]. However, it also appears to have additional functional and ecological implications. In plants, it has been reported to be a relevant nutrient source, especially in conditions of low phosphate availability [15]. At plant root level, exDNA was found on mucilage surrounding the root tips, with a putative protective role [19,20,21] and it has been also shown to act as a signalling molecule, in association with altered expression of specific hormone genes [22]. 

The involvement of exDNA in signalling and self-recognition has been recently widely discussed for plants [1,7,9,23] and in the context of microbe- or damage-associated molecular patterns [24,25,26]. Moreover, the role and the cellular and molecular mechanisms underlying plant growth inhibition determined by extracellular self-DNA (i.e., DNA from the same or closely related species), as well as plant responses to extracellular nonself-DNA (i.e., DNA from phylogenetically unrelated species) are still poorly known and understood. Recent studies on the early plant response to fragmented exDNA [5] revealed a significant plasma membrane depolarization and an increased flux of intracellular calcium in Lima bean (*Phaseolus lunatus*) and maize (*Zea mays*) leaves after treatment with self-DNA, whereas nonself-DNA was unable to trigger such signalling events, thus confirming that plant responses to exDNA depends on the provenience of the DNA also at cellular level. More recently, [27], treating plants and suspension-cultured cells of common bean (*Phaseolus vulgaris*) with fragmented extracellular self-DNA, observed leaf generation of H_2_O_2_, activation of a mitogen-activated protein kinase (MAPK) and increase of extrafloral nectar secretion, that the authors commented as early immunity-related signalling responses. By contrast, nonself-DNA by lima bean and *Acacia farnesiana* exerted lower or no detectable effects. In analogy with mammals, that sense self- or nonself-exDNA as indicators of injury or infection, respectively [28], it was suggested that extracellular self-DNA acts as a damage-associated molecular pattern (DAMP) also in plants [27]. A growth inhibitory effect by self-DNA was demonstrated after a minimum of 48 h [1] and observed up to four days [27] after treatments, while immunity-related signals were observed up to 2 h [5,27]. Therefore, whether growth inhibition by self-DNA depends on the energetic cost of an immunity response [26], or it is a direct effect of the exposure to self-DNA [7], remains an open question. In addition, responses at the cellular level along the timeline preceding observable growth inhibition also deserve further clarification. 

In this study, we analysed early effects after exposure to extracellular self- and nonself-DNA in the plant model *Arabidopsis thaliana* by whole-plant transcriptome profiling, exploiting the RNA-seq approach during the first 16 h post treatment (hpt), and studied the early exDNA spatial distribution at root and cell levels by microscopy confocal analysis. In order to avoid any confounding effect coming from genomic similarity, we used the DNA of the animal *Clupea harengus* (common herring) as nonself treatment, i.e., a species phylogenetically very dissimilar to Arabidopsis.

In this context, specific questions and hypotheses addressed in this work are:

1—What are the early molecular responses to self-DNA before the inhibitory effect on root growth becomes evident?

2—Are such molecular responses different when compared to those from nonself-DNA?

3—Do extracellular self-DNA or nonself-DNA trigger a DAMP or another response?

4—Does early transcriptome evidence support the immunity-cost hypothesis and/or a direct mechanism triggered by self-DNA that exerts an inhibitory effect?

## 2. Results

### 2.1. Differential Gene Expression after Self and Nonself-DNA Exposure

**Figure 1:**
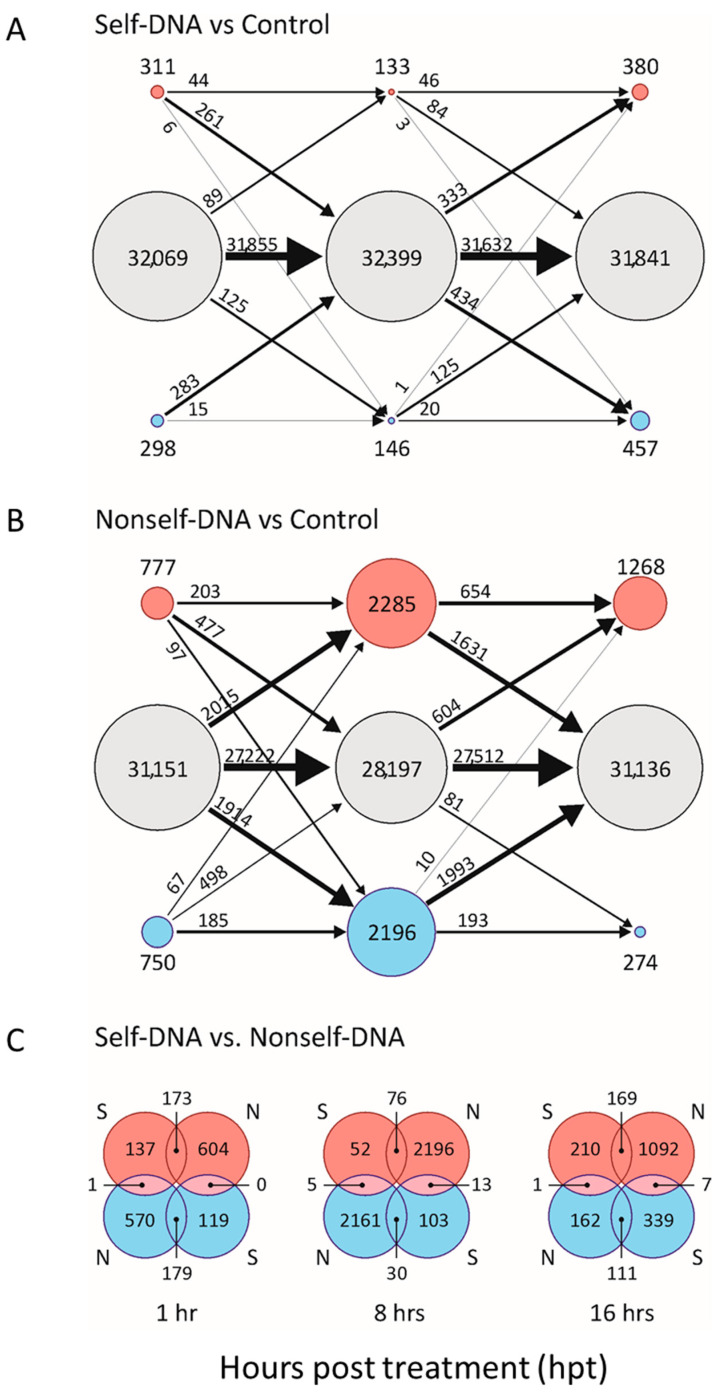
Number of differentially expressed genes (DEGs) (red circles: upregulated; blue circles: downregulated, grey circles: not differentially expressed; circle size log-proportional to gene numbers) in treatment with self-DNA (**A**) and nonself-DNA (**B**) vs. control comparisons and transitions per stage (1, 8 and 16 hpt). The arrows indicate transitions over exposure time (line width log-proportional to the number of transitions displayed on each arrow). In (**C**), the Venn diagram for the self vs. nonself comparisons at each observation stage is reported.

The bioinformatics data processing (Table S1), revealed that, among the 32,678 genes reported in the reference Arabidopsis annotation file, 1473 and 5977 are differentially expressed genes (DEGs) in self and nonself treatments, respectively (Table S2a). Interestingly, the relative number of DEGs in common at each hpt is higher after the first hour than in the other two stages post treatment, with even more striking difference in the number of specific DEGs along the timeline following the exposure to DNA (Table S2b, Figure 1). Indeed, a very limited number of genes are differentially expressed compared to the control after exposure to self-DNA (always less than 2.5% of the total number of Arabidopsis genes). In nonself treatments, the number of DEGs is much higher, especially after 8 hpt, with expression shifts involving more than 15% of the total number of genes. The Venn diagrams of DEGs at each observation stage (Figure 1C) showed that most DEGs were specific per treatment.

After filtering by |log_2_(FC)| ≥ 1, the total number of DEGs was reduced to 825 and 1949 in self and nonself treatments, respectively. Only 342 genes were in common between the two treatments (Table S2a), underlining that specific changes were indeed about threefold higher in nonself treatments than in self ones. Interestingly, the response to self-DNA involved 63% of the filtered DEGs which are specific, i.e., they do not appear in the list of DEGs responsive to nonself-DNA in all the stages.

The complete list of all DEGs and their fold changes, highlighting the filtered DEGs (|log_2_(FC)| ≥ 1) and the expression levels (RPKM) at the corresponding hpt are reported for both self and nonself treatments (Table S3a and b, respectively).

### 2.2. GO Enrichment in Self and Nonself Treatments

**Figure 2:**
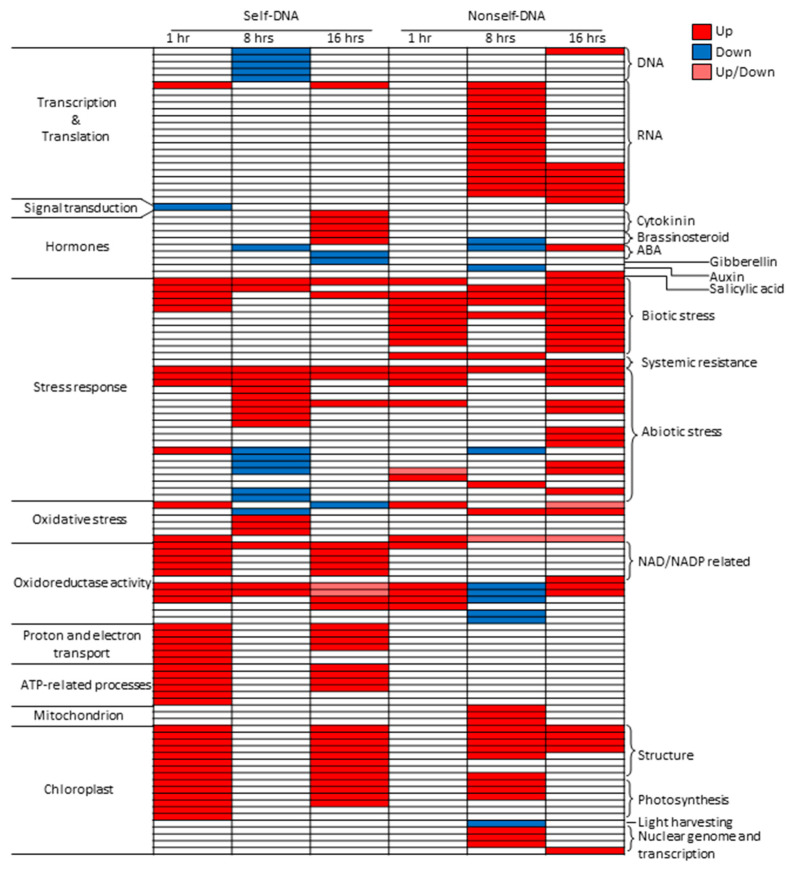
Summary of the GO enrichment analysis on filtered DEGs, with most enriched GOs (rows) grouped by functional process or cell compartment. The colour of each cell in the columns (indicating treatment type and stage) shows the pattern of expression of the enriching genes (full red: upregulated DEGs; blue: downregulated; light red: both up- and downregulated, with enrichment in upregulated DEGs showing lower *p*-value compared to the downregulated ones). In white, absence of enrichment is shown.

Results of gene ontology (GO) enrichment analysis highlighted clear differences across treatments and exposure times for specific classes of GOs (Figure 2, Table S4). Major differences occur in GOs related to DNA transcription and RNA translation, the former showing significant enrichments in downregulated genes after 8 h of exposure exclusively in self-DNA, whereas the group of RNA translation GOs is enriched in upregulated genes at 8 and 16 h exclusively in nonself. At 1 h, and only in self treatments, one GO associated with signal transduction (GO:0007165) is enriched in downregulated genes. GOs related to hormones show relevant differences between self and nonself treatments at 16 h, with GOs related to brassinosteroids and cytokinins enriched by upregulated genes and abscisic acid (ABA) and gibberellin ones enriched by downregulated genes in self-DNA treatment. Nonself-DNA, instead, enriched GO:009751 (response to salicylic acid) by upregulated genes. On the other hand, the nonself-DNA at 8 h shows Brassinosteroids homeostasis, a response to ABA, and Auxin efflux GOs enriched by downregulated genes (Table S4).

GO enrichments by upregulated genes in the group Biotic stress is evident in nonself compared to self-DNA treatments, including the Induced systemic resistance GO term (GO:0009682), enriched at 1 and 8 h, and the systemic acquired resistance (GO:0009627), that is enriched at 16 h exclusively in the nonself-DNA treatment.

The Abiotic stress GO group also shows remarkable differences. In particular, exclusively in self-DNA treatment at 8 h, GOs related to responses to copper and cadmium ions (GO:0046688 and GO:0046686, respectively), as well as to ozone (GO:0010193) and light intensity (GO:0009642), are enriched by upregulated genes. This specificity is held in GOs related to the responses to water deprivation (GO:0009414), high light intensity (GO:0009644), heat (GO:0009408) and hyperosmotic salinity (GO:0042538), as well as to chitin and wounding (GO:0010200 and GO:0009611, respectively). These are all enriched by downregulated genes exclusively in self-DNA treatments (Figure 2, Table S5).

Interestingly, within the oxidative stress GOs group, an enrichment by upregulated genes, involving the response to superoxide radical activity (GO:0019430, GO:0006801 and GO:0004784), and by downregulated genes for the response to hydrogen peroxide (GO:0042542), is evident at 8 h exclusively in self-DNA treatment. A different pattern of enrichment by upregulated genes at 1 and 16 h in self-DNA treatments emerges for the GOs in the groups of proton and electron transport, ATP related processes, and Oxidoreductase activity (Figure 2, Table S4). Noticeably, the latter is instead enriched by downregulated genes in nonself treatments at 8 h.

It is noteworthy that the chloroplast related GOs groups (structure and photosynthesis) show a similar pattern due to enrichments by upregulated genes at 1 and 16 h in self-DNA treatment, while enrichment by upregulated genes at 8 h is evident for nonself-DNA treatments in the case of photosynthesis. Remarkably, light harvesting and the genome and transcription groups within these chloroplast related GOs do not show significant enrichment in self-DNA treatment, while being enriched in nonself-DNA treatments. Interestingly, also mitochondrion related GOs are enriched by upregulated genes exclusively at 8 h in nonself-DNA treatments. 

### 2.3. Functional Response by Multivariate Analysis

**Figure 3:**
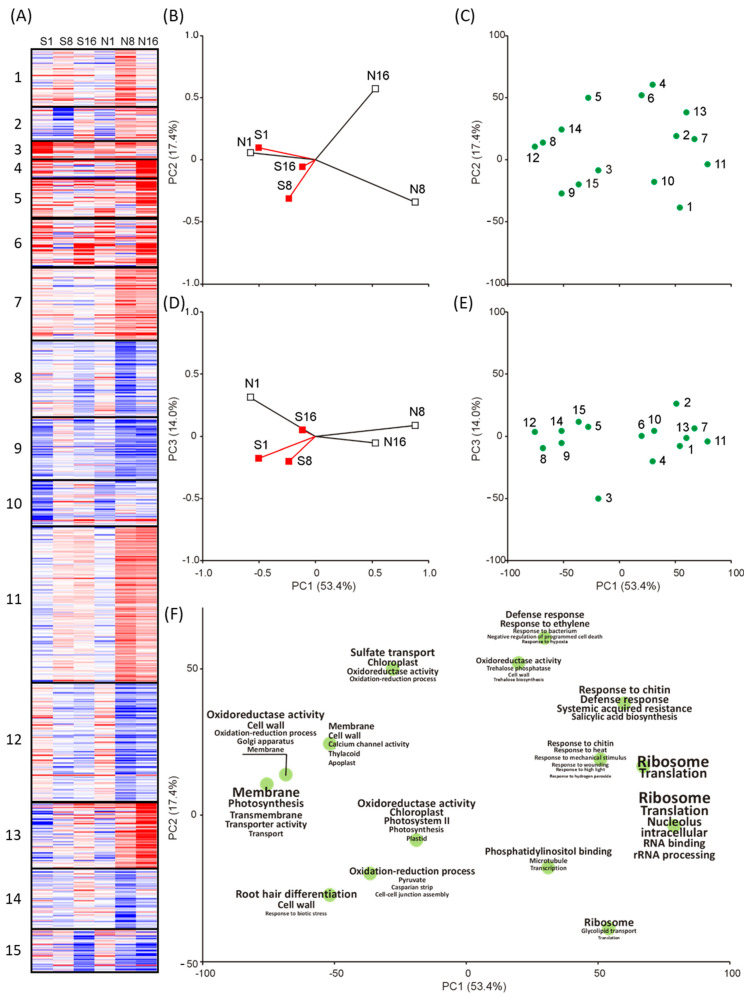
Classification and ordination of gene transcripts and samples based on RNA-seq data. Panels refer to heat map and K-means clustering based on fold changes in all stages of genes resulting differentially expressed (DEGs) in at least one of the six treatments (2 DNA sources × 3 observation times) (**A**). From red (high) to blue (low) the level of fold change values is shown. PCA ordination, with loading vectors of the samples (**B**,**D**) labelled by treatment (S: self-DNA, N: nonself-DNA) and time (1, 8, and 16 h), or factorial scores of cluster centroids (**C**,**E**) is shown. In (**F**), word cloud plot showing the position of each cluster in the first and second principal component space (as in (**C**)), overlapped by the keywords more frequently occurring in enriched gene ontologies (font size inversely log-proportional to enrichment *p*-value).

K-means classification and Principal Component Analysis (PCA) ordination on all DEGs across experimental treatments showed 15 clusters that are clearly separated according to upregulation and downregulation patterns in the experimental treatments (Figure 3A). Details on DEGs in each cluster are reported in Table S5. In the PCA results (Figure 3B–E), the spreading of treatments and clusters in the bi-dimensional space defined by the first three principal components, highlighted several differences according to type and timing of exposure. Indeed, the early response to both treatments are located at the leftmost of the first component (accounting for 53.4% of the total variability), while the samples exposed to nonself-DNA at 8 and 16 h are separated from all other samples at the rightmost of the first component, and from each other along the second component (accounting for 17.4% of the total variability, Figure 3B). The third PCA axis (accounting for 14.0% of the total variability) is related to initial differences between gene expression patterns (at 1 h) in the response to self- versus nonself-DNA treatments (Figure 3D). Plotting the factorial scores of gene cluster centroids (Figure 3C,E) allows the identification of the genes most characterizing the treatments in the same component space (Figure 3B,D). Then, each centroid in Figure 3C is shown in Figure 3F labelled by the most frequently occurring GO keywords in the corresponding cluster (Table S5). Comparing the cluster ordination of Figure 3F with that of treatments in Figure 3B allows to highlight functional response differences among treatments and exposure times. In particular, two major groups of keywords clearly appear along the first principal component. On the left, oxidation–reduction processes and membrane/cell wall contribute to self-DNA treatment at all exposure times, and to nonself-DNA treatment at 1 h, consistent with gene upregulation in these treatments (clusters 8 and 12 in Figure 3A). On the opposite side, at the rightmost of the axis, ribosomal activity mostly contributes to nonself treatment at 8 h, associated with gene upregulation (see clusters 7 and 11 in Figure 3A). Along the second component, a clear distinction within nonself-DNA treatment, between 8 and 16 h, becomes evident, characterized by a defence response, in response to chitin, and a response to bacterium. Such a pattern, indicating a general biotic stress activity, corresponds to significant gene upregulation (clusters 4, 6 and 13 in Figure 3A) at 16 h in nonself-DNA treatment.

### 2.4. Differentially Expressed Genes Associated to Enriched GOs

**Figure 4:**
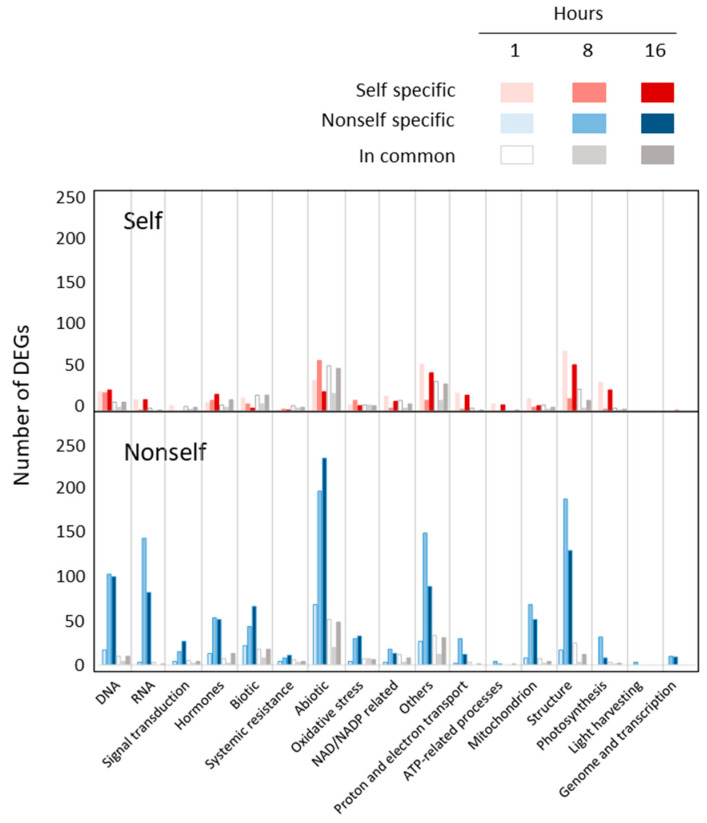
Number of either specific or common filtered DEGs in the main GO groups according to different self- and nonself-DNA treatments (at 1, 8 and 16 hpt). Specific DEGs after exposure to self-DNA are mostly shown at 1 and 16 hpt, whereas the nonself-DNA treatment is associated with an increased number of DEGs at 8 and 16 hpts.

The contribution at gene level of the trends highlighted by the GO enrichment analysis, was analysed in terms of number of DEGs in different GOs according to self- and nonself-DNA treatments (Figure 4). Additional details on up and downregulated DEGs are reported in Supplementary materials (Table S6a,b).

The analysis of DEGs within the GO groups highlighted the different responses between self- and nonself-DNA treatments (Figure 4). The number of specific DEGs after the treatment with self-DNA was higher at 1 h while decreasing at 8 h and rising again at 16 h. In particular, in this treatment, DEGs were observed enriching groups of DNA, RNA, proton and electron transport, ATP-related processes, NAD/NADP related processes and chloroplast related GOs (i.e., structure and photosynthesis) (Figure 4). Moreover, some other GOs (those related to biotic, abiotic stresses and mitochondrion) show a different pattern with a higher number of specific DEGs at the first hour, then progressively decreasing over time at 8 and 16 hpt.

On the contrary, after the nonself-DNA treatment, the number of specific DEGs increased at 8 hrs and persisted also at16 hpt for all considered GOs, with an evident increase in DEGs numbers for DNA, RNA, abiotic stress and chloroplast structure GOs.

#### 2.4.1. One Hour Post Treatment

In the group of GOs related to DNA transcription and translation, the first hour does not report specific enrichments. However, DEGs counting show that downregulated genes prevailed, and the involvement of specific genes per each treatment highlights different reprogramming of the transcriptional asset in the two treatments. Interestingly, among the 12 upregulated genes in the self-DNA treatment, we identified the four RNA polymerases encoded by the chloroplast genome (ATCG00170; ATCG00180; ATCG00190; ATCG00740) all expressed over the log_2_(FC) > 1 cut-off (Table S3). In addition, self-specific upregulated DEGs include AT1G66600 (ABO3), a WRKY transcription factor involved in drought tolerance and in ABA mediated response [29]; AT5G47220 (ERF2), the ethylene responsive element and two transcription factors that are involved in ROS response (AT3G46080, AT1G52890). Noticeably, the higher expression of ERF2 is accompanied by the downregulation of specific genes involved in ethylene signal transduction (AT4G34410, AT5G52020) and ethylene responsive transcription factors (AT1G74930, AT1G28370). AT3G01220, exclusively downregulated in self-DNA treatment, is expressed during seed germination in the micropylar endosperm and in the root cap, and when mutated, increases seed dormancy and ABA sensitivity [30]. 

We identified 13 DEGs in response to self-DNA treatment associated with the enriched GOs among the RNA related GOs in the Transcription and translation group. All genes are encoded by the chloroplast genome (Table S3), with the only exception of one mitochondrial genome encoded gene (ATMG00090) that codes for a ribosomal protein S3 (RPS3), which is reported to contain a domain for resistance to pseudomonas syringae 3.

The GO:0007165 (signal transduction) was enriched by downregulated genes in self-DNA treatment, and the contribution of 5 specific genes encoding a CBL-interacting protein kinase (CIPK25) (AT5G25110), two Toll-Interleukin Resistance (TIR) proteins (AT5G44910, AT5G44920), an ankyrin repeat related gene (AT4G0346) and a phloem protein 2 A5 (PP2-A5) (AT1G65390), that is known to be induced upon and confers tolerance to spider mite attack [31] (Table S3).

Considering the 6 GOs NAD/NADP related processes in the group of oxidoreductase activity, 14 genes are exclusively upregulated in response to self-DNA treatment, of which 10 are encoded by the chloroplast genome (Table S3). 

The group of proton and electron transport GOs included 6 GOs, with 21 genes exclusively upregulated in response to self-DNA treatment (Table S6a). These genes are mainly involved in the photosystem II reaction centre, in photosynthetic electron transfer and in the ATP synthase complex or membrane transporters. It is worth noting that when considering the GO group of ATP related processes, all the DEGs are self-specific and code for the ATP synthase complex, and all these genes are also contributing to the enrichment of the group of proton and electron transport related GOs.

Although the lack of enrichment in the GOs related to Mitochondrion in the first hour post both treatments, there are DEGs associated with these GOs. Considering the specific DEGs in each treatment, a higher contribution by self-DNA-related DEGs is reported (Table S6a). Interestingly, among these genes AOX1d (AT1G32350) encodes for one of the 4 AOX1 genes of *A. thaliana* genome [32].

When considering the chloroplast related GOs, where all the 4 groups of GOs are exclusively enriched in upregulated genes in the self-DNA treatment, the 7 GOs related to Structure (represented by a total of 2541 genes, Table S6a), reveal that the DEGs in response to self-DNA contributed more than the nonself treatment (94 versus 42 genes, and 69 versus 17 specific, respectively) (Figure 4). The major contribution was from upregulated genes (88 and 33 in self- and nonself-DNA treatments, respectively). Among the 94 DEGs in response to self-DNA treatment, 61 genes are encoded by the chloroplast genome and 57 of these chloroplast genes are specific of the self-DNA response at this stage. 

In the case of the photosynthesis group (36 DEGs out of a total of 199 genes) all DEGs were from the self-DNA treatment (Figure 4), with 35 upregulated genes and 32 of them specific to this treatment, and 34 genes over 35 encoded by the chloroplast genome. In the nonself-DNA treatment, out of a total of 3 DEGs, 2 were upregulated and nonspecific DEGs (Table S6a).

The biotic stress GOs included 11 GOs associated with a total of 490 genes (Table S6a). These GOs were associated with 27 (10 specific) and 6 (5 specific) upregulated and downregulated genes, respectively, in self DNA response, while the corresponding figures for the nonself-DNA treatment were 40 (total DEGs), 34 (17 specific) upregulated and 6 (5 specific) downregulated DEGs, respectively, thus indicating remarkable difference in the response between the two treatments (Figure 4). Interestingly, among the DEGs, the self-DNA treatment shows a specific upregulation of BAG6 (AT2G46240) [33], one of the three genes belonging to the BAG family (AtBAG4, AtBAG6 and AtBAG7) that controls the induction of autophagy and have confirmed cytoprotective activities in response to cold, drought and heat stress. 

The 2 enriched GOs within the Systemic resistance group corresponded to a total of 60 genes, 11 of which were DEGs at 1 h (Table S6a). PAD 4 (AT3G52430: phytoalexin deficient 4), that usually mediates TIR-NB-LRR signalling involved in the pathogen resistance response, as well as in root meristem growth arrest [34], is exclusively downregulated in self-DNA treatment and this is also confirmed by QRT-PCR (Table S7).

Considering the group of GOs associated with abiotic stress (20 enriched GOs for a total of 2697 genes), we identified 66 (24 specific) and 21 (11 specific) DEGs among upregulated and downregulated genes, respectively, in the self-DNA treatment. A total of 44 genes are upregulated and specific, while 35 are downregulated—25 of which are specific—in the nonself treatment, again sowing the differential responses between the two treatments (Table S6a, Figure 4). 

In the group of oxidative stress (6 GOs for a total of 204 genes), 2 GOs were enriched at 1 h, corresponding to a total of 19 DEGs. Most of the genes (13 out of 19) in the self-DNA treatment are also included in DEGs involved in the abiotic stress GOs, indicating that the oxidative stress contribution is a key component of the early response elicited by self-DNA (Tables S3 and S6a). 

#### 2.4.2. Eight Hours Post Treatment

DEGs associated with the group of DNA enriched GOs, correspond to 25 and 108 total DEGs in self- and nonself-DNA treatments, respectively (Table S6a). Five genes are upregulated in response to self-DNA treatment at the 8 h (with 1 specific gene) and 65 (61 specific) are upregulated in response to nonself-DNA. All downregulated genes, 20 and 43 in response to self- and nonself-DNA, respectively, were specific of each treatment. Interestingly, although a higher number of DEGs is reported in nonself-DNA treatment, an exclusive GO enrichment (from downregulated genes) is evident in the self-DNA treatment, hence depicting a clear trend in the self-DNA response at 8 hpt (Figure 4). Among the genes that are downregulated in the self-DNA treatment, many follow the same trend already evident in the 1 hpt (Table S3). Among these genes, HSFA2A (AT2G26150), a transcription factor that is typically upregulated during stress response [35], and HSFC1 (AT3G24520), both confirmed by QRT-PCR, together with other AT-HSFA7B (AT3G63350) and 3 heat shock proteins (HSPs), all showing a significant negative log_2_(FC) in self-DNA treatments. The majority of the downregulated genes are ethylene related proteins or ethylene responsive transcription factors (AT1G12610: dwarf and delayed flowering 1 (DDF1); AT1G19210: Integrase-type DNA-binding superfamily protein; AT1G74930: ORA47; AT2G44840: ethylene-responsive element binding factor 13 (ERF13); AT4G11280: 1-aminocyclopropane-1-carboxylic acid synthase 6 (ACS6); AT4G25490:C-repeat/DRE binding factor 1 (CBF1); AT4G34410: redox responsive transcription factor 1 (RRTF1); AT5G05410: DRE-binding protein 2A (DREB2A); AT5G52020: Integrase-type DNA-binding superfamily protein; AT4G28110: myb domain protein 41 (MYB41)). In contrast, among the DEGs, those associated with salicylic acid or to the activation of the systemic acquired resistance highlight remarkable differences at transcription level between self- and nonself-DNA treatments at 8 h.

Considering the group of GOs associated with RNA, 18 GOs (related to cytoplasmic translation, biogenesis and/or assembly of ribosome and its subunits, rRNA cleavage, methylation and processing, rRNA binding) were enriched in upregulated genes exclusively in nonself-DNA treatment. Among the 146 DEGs, 145 genes were all specific to the nonself-DNA treatment, with 141 upregulated genes, among which 49 encode ribosomal proteins, indicating a consistent activation of the translation machinery in the nonself-DNA treatment at this stage (Table S6a, Figure 4). 

Considering signal transduction, the reported GO:0007165 that is significantly enriched at 1 h, is not enriched at 8 h. However, at this stage we identified 17 DEGs associated with this GO (Table S6a, Figure 4), out of which only 2 genes are DEGs (upregulated) in response to self-DNA, both in common with the nonself-DNA treatment (AT4G11170 (Disease resistance protein (TIR-NBS-LRR class) family) and AT5G44990 (Glutathione S-transferase family protein)). To be noted, among the 13 upregulated DEGs in nonself-DNA treatment (Table S6a), 6 genes (AT1G57630, AT1G66090, AT1G72900, AT4G10170, AT5G41750, AT5G45000) are TIR domain containing proteins. Four downregulated genes were all specific in nonself-DNA treatment. A couple of them are involved in the signalling of phosphoinositides: AT3G55940 (Phosphoinositide-specific phospholipase C family protein) and AT5G58670 (phospholipase C1 (PLC1)). Interestingly, among the significant downregulated genes in nonself-DNA we also identified 4 further genes (AT3G03530: non-specific phospholipase C4 (NPC4); AT3G08510: phospholipase C 2 (PLC2); AT3G48610: non-specific phospholipase C6 (NPC6); AT3G51460: ROOT HAIR DEFECTIVE4 (RHD4)) all involved in the phosphoinositides signalling pathway, which is recognized as an early response involving membrane reorganization and lipid signalling in defence response [36]. 

In the group of biotic stress, we identified a total of 16 DEGs in the self-DNA treatment, 12 (4 specific) upregulated and 4 (all specific) downregulated (Table S6a). Among the upregulated genes, AT3G26830 (PHYTOALEXIN DEFICIENT 3 (PAD3)), AT5G40990 (GDSL lipase 1 (GLIP1)) and AT1G79680 (WALL ASSOCIATED KINASE (WAK)-LIKE 10 (WAKL10)) resulted upregulated at all observation stages in both DNA treatments, with the exception of the nonself-DNA treatment at 8 h. Differently, the self-specific downregulation of AT2G46240 (BAG6), AT1G80840 (WRKY40), AT2G27080 (Late embryogenesis abundant (LEA) hydroxyproline-rich glycoprotein family) and AT2G40000 (ortholog of sugar beet HS1 PRO-1 2 (HSPRO2)), putatively indicates a decrease of the biotic response to pathogen [37]. Accordingly, plants exposed to self-DNA showed a negligible representation of differential expression from biotic stress genes, when compared to abiotic stress related ones (Table S6a, Figure 4). Considering the GOs related to the systemic acquired resistance, we identified 13 DEGs, out of which 5 in the response to self-DNA (4 upregulated genes of which 2 specific), and 9 upregulated genes (7 specific) in the response to nonself DNA (Table S6a). Among these, AT4G12470 (AZI1), which is involved in the priming of salicylic acid induction and systemic immunity [38], was downregulated at 1 h and upregulated at 8 h in the nonself-DNA treatment. 

Within the group abiotic stress, we found 78 DEGs in response to self-DNA treatment, out of which 35 were upregulated (18 specific), and 43 downregulated (40 specific) genes (Table S6a). In the case of nonself-DNA treatment, out of a total of 219 genes (Figure 4), 129 were upregulated (110 specific) and 90 downregulated (80 specific) genes (Table S6a). Among these genes, AT3G48360 is downregulated in self and is upregulated in nonself-DNA treatment. This gene has been shown to be downregulated in the presence of sugar while it is upregulated in the presence of nitrogen [39]. Moreover, the 35 upregulated genes in response to self-DNA included 5 GST genes (2 of which specific) and 5 peroxidases (1 specific), while among the 40 genes specifically down expressed in self-DNA treatment, 18 belong to the HSP protein superfamily (Table S3). 

In the group of oxidative stress, we found 19 DEGs in self-DNA treatment, 6 of which upregulated (2 specific), and 13 were downregulated (10 specific) (Table S6a, Figure 4). Among the 2 genes exclusively upregulated in response to self-DNA, AT4G25100 codes for Fe superoxide dismutase 1 (FSD1), acting in plastidial, cytoplasmic and nuclear compartments with an anti-oxidative and osmoprotective role [40]. 

In the group of oxidoreductase activity, in the GOs concerning NAD/NADP related processes, we identified a total of 6 DEGs in the self-DNA treatment, all upregulated and 3 specific, while in the nonself-DNA treatment we found 15 upregulated genes (12 specific) and 6 downregulated genes, all specific (Table S6a). 

In the group of proton and electron transport (Figure 4), we found a total of 32 DEGs, out of which 2 were differentially expressed in response to self-DNA (downregulated in this treatment), and 30 DEGs, with 17 and 13 specific genes upregulated and downregulated, respectively, in the nonself DNA treatment (Table S6a). 

In the group of ATP-related processes, only 4 genes were differentially expressed, all exclusively in nonself-DNA treatment (Table S6a, Figure 4).

In the group of mitochondrion related GOs (3 GOs for a total of 1411 genes) we found 75 DEGs (Table S6a). It is noteworthy that among the three genes exclusively downregulated in response to self-DNA, AT4G25200 encodes for a mitochondrial localized small HSP that is regulated by HSFA2, that, as mentioned above, is also downregulated in the self-DNA treatment [41]. Differently (Figure 4), in nonself-DNA treatment, a total of 71 DEGs included 59 (57 specific) and 12 (all specific) upregulated and downregulated genes, respectively (Table S6a, Figure 4). Interestingly, one gene showed opposite behaviour in the two DNA treatments, being downregulated and upregulated in response to self- and nonself-DNA, respectively. This gene encodes for HSP26.5 (AT1G52560) that also is correlated in its expression with HSFA2 [41].

It is worthy to note that in the GO group of chloroplast (including structure, photosynthesis, light harvesting, and genome and transcription) no GO was enriched in self-DNA treatment at 8 h (Figure 2). Nevertheless, we still found specific responses in the self (17 DEGs including 10 (8 specific) upregulated and 7 (6 specific) downregulated genes) and in the nonself-DNA treatment (144 DEGs (142 specific) were upregulated and 49 (48 specific) were downregulated (Table S6a). 

#### 2.4.3. Sixteen Hours Post Treatment

After 16 hpt, in the group of DNA GOs, 135 genes are DEGs in self- and nonself-DNA treatments (Table S6a), 14 up and 10 down regulated genes are specific of the self-DNA treatment, while 94 up and 7 down regulated genes are specific of the nonself-DNA treatment (Figure 4). Among the 94 nonself- specific and upregulated genes, 13 belong to the MyB transcription factors family and 22 are WRKY transcription factors. Interestingly, among these latter genes, WRKY33 is involved in both the hypersensitive response and the systemic acquired resistance and WRKY70 is involved in the establishment of the systemic acquired resistance [42]. This indicates that nonself-DNA can be sensed as a PAMP by triggering the hypersensitive response and initiating a systemic acquired resistance.

In the group of RNA GOs, 97 were the total DEGs, including 14 DEGs in self-DNA treatment, 13 of which (12 specific) are upregulated, and 1 downregulated and specific, while 83 specific among 84 upregulated genes are DEGs in the nonself-DNA treatment (Table S6a, Figure 4). Interestingly, the 12 genes exclusively upregulated in response to self-DNA are encoded by the chloroplast genome and 8 of them are also upregulated at the 1 h post the same treatment. Differently, in the nonself-DNA treatment, the upregulated genes are more related to rRNA processing, maturation and stabilization, and less to ribosome biogenesis and structure (Table S3).

In the group of signal transduction GOs, with 1 GOs for a total of 432 genes (Table S6a), we report 31 total DEGs, all upregulated in nonself-DNA treatment (27 specific and 4 in common with the self-DNA treatment) (Table S6a, Figure 4). Among the 27 genes exclusively upregulated in response to nonself-DNA, 12 belongs to the group of disease resistance proteins (Table S3) and appear to be involved in the process of the hypersensitive response [43].

Concerning the stress response class of GOs, the group of biotic stress includes 88 DEGs in total all upregulated, with 21 DEGs (3 specific) in self and 85 DEGs (67 specific) in nonself treatments, respectively (Table S6a, Figure 4).

The group of systemic resistance showed a total of 16 DEGs, with 5 DEGs in self-DNA treatment (3 upregulated and in common with nonself, and 2 downregulated and one in common with nonself), and 15 genes, 13 (10 specific) upregulated and 2 (1 specific) downregulated in nonself (Table S6a). Among the 10 genes exclusively upregulated in response to nonself-DNA, AT3G52430 encodes for PAD4 which, as discussed above, when upregulated, mediates the TIR-NB-LRR signalling involved in the hypersensitive response [43].

In the group of abiotic stress, we identified 308 DEGs. Most of them (252), among which 213 specific DEGs, are upregulated in response to nonself-DNA, while 34 (24 specific) are downregulated in this treatment. In the self-DNA treatment, 51 genes (12 specific) are upregulated and 20 (10 specific) are downregulated (Table S6a, Figure 4). 

In the group of oxidative stress, we found a total of 45 DEGs, with 12 total DEGs in response to self-DNA, including 4 (1 specific) upregulated and 8 (5 specific) downregulated genes, and 39 DEGs in nonself-DNA treatment, including 30 (27 specific) upregulated and 9 (6 specific) downregulated genes (Table S6a, Figure 4). In particular, 13 out of the 27 genes exclusively upregulated in response to nonself-DNA are peroxidases (Table S3). 

Concerning the oxidoreductase activity GOs for the group of NAD/NADP related processes GOs, we identified a total of 32 DEGs. Considering the 10 DEGs exclusively upregulated in response to self-DNA treatment, 8 of them, coding for NADH dehydrogenase subunits, are encoded by the chloroplast genome and resulted upregulated also at 1 h (Table S3). In the nonself-DNA treatment, out of 21 DEGs, 17 (9 specific) were upregulated and 4 (all specific) were downregulated. Among the 9 genes exclusively upregulated in response to nonself-DNA treatment, none is encoded by the chloroplast genome, and 7 belong to the cytochrome p450 protein family. 

In the group of proton and electron transport, we found 31 DEGs in total, with 19 DEGs, including 18 (17 specific) upregulated genes and 1 specific and downregulated gene, in self-DNA treatment (Table S6a). Among the 17 genes exclusively upregulated in response to self-DNA treatment, 16 are encoded by the chloroplast genome and all were also upregulated at 1 h. 

For the group of ATP-related processes, exDNA treatments show nine DEGs in total. All of these are upregulated DEGs (Table S6a), including eight (seven specific) and two (one specific) DEGs in response to self- and nonself-DNA, respectively. Among the seven genes exclusively upregulated in the self-DNA treatment, six are encoded by the chloroplast genome and all are upregulated also at 1 h (Table S3). 

For the group of mitochondrion, treatments showed a total of 42 DEGs, with 54 (50 specific) upregulated and 2 (all specific) downregulated genes in nonself-DNA treatment, and 8 upregulated (4 specific) and 2 downregulated and specific genes in response to self-DNA, the latter including AT5G24120 (Table S3). It is worth noting, such gene codes for SIGE, a transcriptional factors localized in both chloroplast and mitochondrion, which regulates the chloroplast transcriptional response to light intensity [44]. 

Considering the chloroplast group of GOs, the self-DNA treatment shows a trend similar to that observed at 1 h for the Structure and Photosynthesis subgroups (Table S6a, Figure 4). In the subgroup of structure, we found 196 DEGs, out of which 55 (46 specific) were upregulated and 10 (7 specific) were downregulated in self-DNA treatment. Among the 46 specific DEGs, 45 are encoded by the chloroplast genome and 40 of them are upregulated also at 1 h (Table S3). In nonself-DNA treatment, out of a total of 143 genes, 136 (127 specific) are upregulated and 7 (4 specific) are downregulated (Table S6a). Different from the self-DNA treatment, among the 127 genes upregulated and specific of the response to nonself-DNA, only 16 were upregulated also at 1 h and, interestingly, none is encoded by the chloroplast genome (Table S3). In the group of photosynthesis, we identified 34 DEGs (Figure 4). In self-DNA treatment, among a total of 26 DEGs, 23 were upregulated (21 specific) (Table S6a). Out of the 21 upregulated and specific genes, 20 are encoded by the chloroplast genome and were upregulated also at the 1 h (Table S3). In nonself-DNA treatment, out of a total of 10 DEGs (Figure 4), 9 were upregulated (7 specific) (Table S6a). For the group of light harvesting no DEGs are evident in both self- and nonself-DNA treatments. 

In conclusion, in the self-DNA treatment, the genes involved in processes related to cell energy production and balance, oxidoreductases and chloroplast structure and photosynthesis show a recursive upregulation, since the pattern at 16 h resembles the one at 1 h.

### 2.5. Hormone Related DEGs after Self- and Nonself-DNA Treatments

The Hormones enriched GOs (10 GOs for a total of 610 genes) indicate processes related to cytokinins, brassinosteroids, ABA, gibberellins, auxins and salicylic acid. 

To consider more details on possible DEGs associated with hormones, we considered the number of DEGs searching by each hormone as a keyword in GO categories of DEGs (Table S6b). 

In the self-DNA treatment, among the upregulated DEGs at 1 h, we found (ranked by counts): nine genes (six specific) in the group of abscisic acid, seven genes, all self-specific, in the group of jasmonic acid (JA), five genes (three specific) for cytokinins, five (one specific) for salicylic acid, four for auxin, and one for the ethylene, while brassinosteroids and gibberellins were not involved in differential expression at this stage. Among these upregulated DEGs, it is worthy to mention AT5G15970, encoding for the protein KIN2, that is known to be induced by ABA and during water deficit stress. Additionally, AT1G15520 (PDR12) encodes for the pleiotropic drug resistance 12, an ABA related ABC transporter localized on the plasma membrane of guard cells and involved in ABA uptake and stomatal closure [45,46]. 

In the case of nonself-DNA treatments, among hormone with upregulated genes, we found: salicylic acid (five specific), abscisic acid (three specific), auxin (two specific), cytokines (one specific), ethylene and brassinosteroids (one specific). Among the downregulated DEGs, six were in the group of ABA (five specific), four in salicylic acid (four specific), three in cytokinins (two specific), two for the ethylene, both DEGs are nonself-specific, one specific in the group of brassinosteroids, and one in the group of auxin. We did not find DEGs for JA and gibberellins, neither among upregulated, nor among downregulated genes for nonself-DNA treatment at this stage. Overall, the data show a relevant role of ABA and JA in the response to self-DNA exposure at 1 h, while, in the case of nonself-DNA treatment, salicylic acid, ABA and auxin, play a major role at 1 h (Table S6b, Figure 4). 

After 8 h of exposure to exDNA, we found a total of 85 DEGs. Among the 16 DEGs found in response to self-DNA, 7 were upregulated (6 specific) and 9 were downregulated (6 specific), whereas in nonself-DNA treatment, out of 73 DEGs in total, 25 were upregulated (23 specific) and 48 were downregulated (46 specific), indicating that the overall hormone reprogramming was active mainly in this specific treatment, at this stage. Hormone-related DEGs counts reveal a reduced involvement of ABA and JA associated DEGs in comparison with the first hour, in the response to self-DNA. In the case of nonself-DNA treatment, a remarkable specificity characterizes the response in comparison with the self-DNA treatment, with a clear involvement of salicylic acid, ABA and auxin activity related DEGs (Table S6b, Figure 4). 

At 16 hpt, a total of 84 DEGs are evident (Table S6b, Figure 4). Among the upregulated genes in the self-DNA treatment, AT4G29740 and AT5G56970 encode for oxidases/dehydrogenases that catalyse the degradation of cytokinins, that are mainly involved in cell division processes and cell growth and differentiation [47], thus possibly revealing effects related to the growth inhibition caused by self-DNA exposure [1] 

The high number of hormone-related DEGs in nonself-DNA treatment at 16 h, compared to the previous observation stages, may indicate a reprogramming of the hormonal activity in the nonself- compared to self-treatments (Table S6b, Figure 4).

A remarkable general difference on number of DEGs in self-DNA exposure when compared with nonself treatments, and also on their trends in different times post treatments is evident. Noticeable, the ABA, jasmonic acid, salicylic acid, ethylene and cytokinins related DEGs, increase in nonself-treatments in total and as specific upregulated DEGs during time, while the same trends are not evident in the self treatments. 

### 2.6. DAMP and PAMP Associated Genes

To consider Arabidopsis genes involved in DAMPs or in PAMPs responses, we collected the list of known or putative receptors, mainly considering those responsive to extracellular nucleic acids, described in the literature [9,48,49,50,51,52,53,54,55,56,57,58,59,60,61,62,63,64]. Moreover, we also considered the expression patterns in both self and nonself-DNA treatments comparing the behaviour per stage (Table S8a). The summary of the total number of DEGs from self and nonself-DNA treatments at different time post exposure is reported in (Table S8b). Interestingly, it is evident that in both treatments, there are DEGs responsive genes in either the DAMP or the PAMP classes (Table S8b). In particular, in the DAMP class, the number of DEGs increases in both self and nonself treatments during time. In contrast, in the PAMP class, the number of DEGs remains almost stable in self treatments, while it is higher in the first and third stages, in comparison with the second stage post treatment, in nonself-DNA treatments. Interestingly, the number of DEGs showing a common behaviour in the two treatments increases during time for both classes, although the number of specific DEGs remains higher in nonself treatments, especially in the DAMP class. This may be due to the increase of DAMPs in the later stages of the nonself treatments, due to the cellular disruption revealed by the confocal analysis. It is worth noting, the very low numbers of specific DEGs from self-DNA treatments in both classes, which is even more striking in the PAMP class, and specifically at the 16 hpt. This may indicate that the differential sensing may be determined in the initial stages post treatment. Nevertheless, from this preliminary analysis, it is evident that the response to self-DNA is poorly characterized in terms of receptors of DAMPs or PAMPs (Table S8b). Considering the DEGs that are specific in the self-DNA treatment (Table S8a), it is worth mentioning AT1G57650, coding for an ATP-binding protein, and AT1G57630, coding for a TIR domain family protein, both upregulated and reported to respond to extracellular nucleic acids [9] and AT1G31540, coding for a TIR-NBS-LRR protein, which is down regulated, all belonging to the DAMP class. AT2G19190, coding for the Flagellin22-induced receptor-like kinase 1 and AT1G02900, coding for a Rapid alkalinization factor are both down regulated in the DAMP class.

At 8 hpt, specific DEGs from self-DNA treatments include defensins (three up regulated and one downregulated), and AT1G79680, coding for a cell wall associated kinase (WAK10), which is upregulated and reported to be a calcium receptor, and AT2G33580, in the PAMP class, coding for another membrane kinase, which is downregulated. Interestingly, among the three defensins that are classified as DAMPs and have DEGs in the nonself response, none is in common with the self-DNA response, and all are downregulated expect the one coded by AT3G24510, that is up regulated. It is of interest to note that the defensin pattern remains almost different in the two treatments also in the third stage. In particular, AT5G33355 remains upregulated also at the 16th hpt in self while AT3G24510 remains up in nonself treatments. AT1G34047 results a DEG at the 16 hpt only in the self-DNA response. Remarkably, defensins are the major class that is involved in the specific self-DNA response among the two classes (Table S8a).

### 2.7. QRT-PCR Results

Table S7 shows the results from QRT-PCR data of seven genes compared with the fold change of DEGs. The selected genes were chosen also to confirm some of the marker genes that could depict the behaviour in the two treatments. It is worth noticing the upregulation of the superoxide dismutase in self confirms the oxidative stress which is typical of this treatment. The expected general trend of AOX1d is confirmed in the two treatments per stage, together with the down regulation of HSFA2 (AT2G26150) and HSFC1 (AT3G24520) in the second stage of the self-DNA treatment, which is even more evident in the nonself one. Expression levels observed by RNAseq and QRT-PCR were well in accordance, as confirmed by the highly significant linear regression between the two series of data emerging from the comparison across seven gene transcripts, two DNA treatments and three observation time points (Figure S1, Pearson’s r = 0.814, P = 1.73 × 10^−10^).

### 2.8. Differential Self- and Nonself-DNA Distribution by Confocal Analysis and Phenotypic Changes in Seedlings

**Figure 5:**
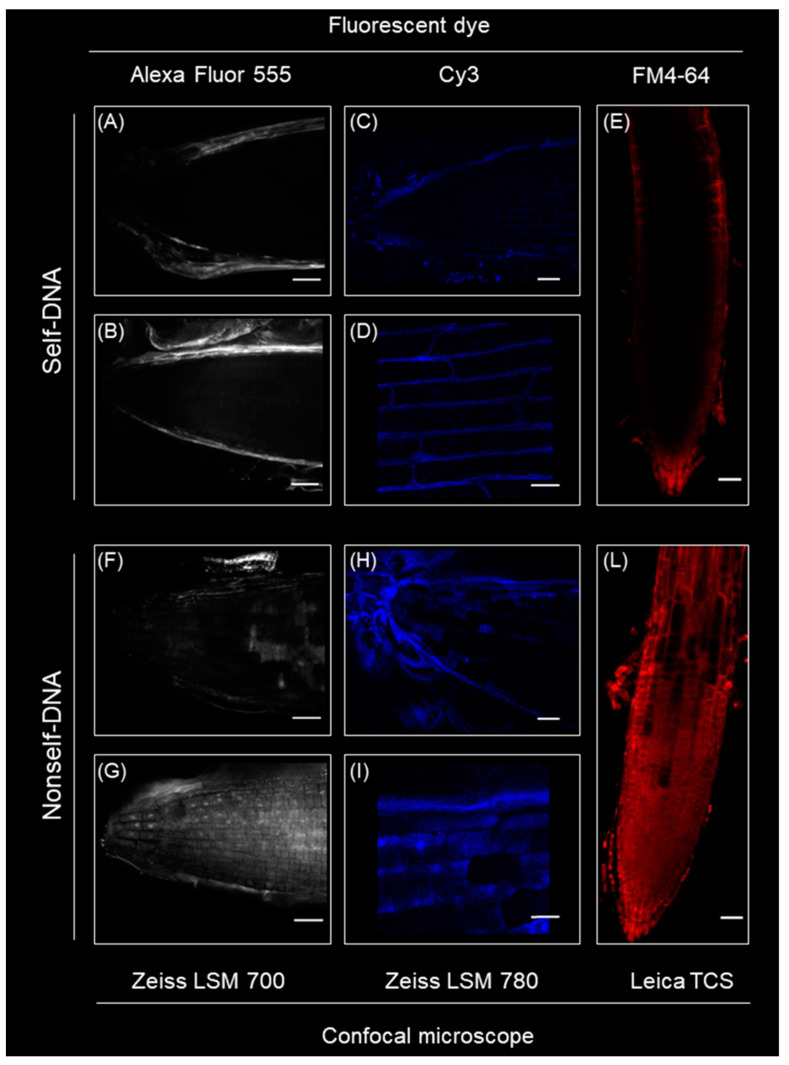
Fluorescence microphotography (confocal images) of early response (1 h) to self- vs. nonself-DNA treatments. From the left to the right, the following treatments are shown: Alexa Fluor 555 dye (in white) labelling self-DNA (**A**,**B**) and nonself-DNA (**F**,**G**); Cy3 dye (in blue) labelling self-DNA (**C**,**D**) and nonself-DNA (**H**,**I**); FM4-64 staining (in red) of A. thaliana roots after 1 h exposure to either self-DNA (**E**) or nonself-DNA (**L**). Scale bars 20 µm in (**A**–**C**,**E**–**G**,**I**,**L**); 10 µm in (**D**,**H**).

Confocal microscopy of *A. thaliana* roots exposed to self-DNA revealed that labelled DNA (with both Cy3 and Alexa Fluor 555 dyes) was mainly visible outside the roots (Figure 5A–D) with an absence of fluorescence in the cytoplasm evident in all the images. At 1 h, the self-DNA fluorescence could be detected inside the root, but limited to the surface of the cells (Figure 5D). 

In contrast, labelled nonself-DNA was clearly uptaken by the roots, in the cytoplasm and even at nuclear level (Figure 5F-–I). The negative control showed no Cy3 fluorescence signal in any part of the root (data not shown). The exposure of the roots to FM4-64 dye after their treatments with either self- or nonself-DNA, showed a striking difference in the dye uptake and diffusive pattern inside the roots according to the type of extracellular DNA. Indeed, the dye remained outside the root that were previously exposed to self-DNA (Figure 5E), whereas it massively entered the root cells when they had been previously treated with nonself-DNA (Figure 5L).

**Figure 6:**
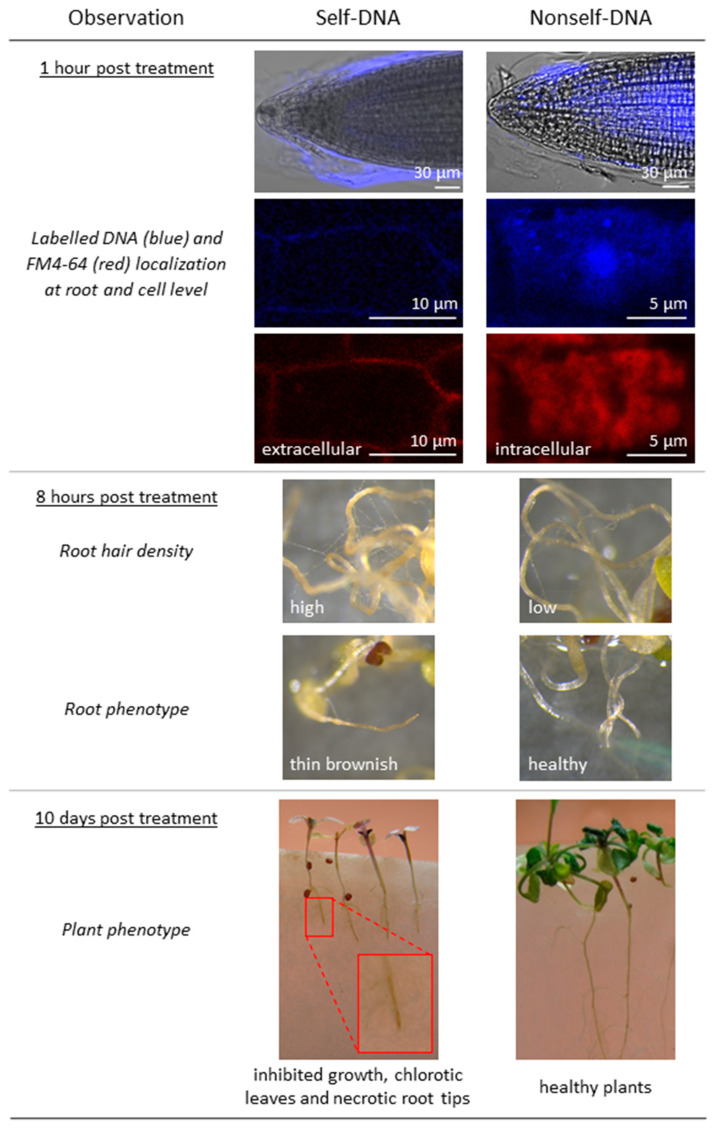
Summary of the main differences in response to extracellular self- and nonself-DNA in plants. A differential uptake according to treatments is clear after 1 h, morphological differences on thin roots and root apices appear after 8 h, full inhibition by the exposure to self-DNA is evident on the whole plants after 10 days.

At the phenotypic levels, the main differential responses to self- and nonself-DNA are summarized in Figure 6. Of note, at macroscopic level, the exposure to self-DNA produced peculiar phenotypic effects: at 8 hpt with self-DNA, there is an increase in root hair density and a consistent root brownish; at a later stage 10 days post treatment, necrosis of root tips is accompanied by an inhibition of growth and leaf decolouring (Figure 6).

## 3. Discussion

In Mazzoleni et al., 2015, it was reported that extracellular self-DNA, released in the soil during litter decomposition, or made available by experimental exposure, induced an inhibitory effect on root growth and seed germination in several plant species, without affecting heterospecifics [1]. Such findings have been ascribed to different putative mechanisms [8], including signalling and self-recognition [9,23], plant root defence [19] and microbe- or damage-associated molecular patterns [24,25,26].

The current study shows a clear-cut pattern in the plant transcriptomic response in the early stages after exposure and before evident phenotypic traits could be detected. Exposure to exDNA resulted in remarkable differences both between exposure to self- vs. nonself-DNA, and among different stages after exposure in each treatment. In parallel, remarkable differences were also highlighted in the early response by confocal microscopy showing that the root treated with self- and nonself-DNA have totally different physiological responses. A reduced root cell membrane permeability appears following the treatment with self-DNA, as indicated by the accumulation of both labelled self-DNA and FM4-64 in the outer layers of root cells. Conversely, after treatment with nonself-DNA, the labelled DNA diffused throughout the root reaching also the nuclei and, in this case, a clear diffusion of FM4-64 in the innermost part of the root was also evident. 

The significantly different patterns of entrance of both labelled-DNA and of FM4-64 dye were consistent with the transcriptomic analysis results showing only in the case of nonself-DNA treatment the establishment of a hypersensitive response associated with cell wall and plasma membrane remodelling [65]. The uptake of nucleic acid macromolecules in roots was already reported [22]. However, to the best of our knowledge, this is the first evidence of exDNA entry in roots showing different distribution patterns between self and nonself-DNA. 

In particular, the evident uptake of FM4-64 only after the exposure to nonself-DNA suggests an interesting activation of processes of endocytosis, vesicle dynamics and organelle organization as already reported in eukaryotic cells and plants [66].

At macroscopic effects peculiar phenotypic effects are revealed post self-DNA treatments, with an increased root hair density and a consistent root brownish evident already at 8 hpt. Only the exposure to self-DNA produced necrosis of root tips, inhibited growth and leaf decolouring at 10 days post treatment.

### 3.1. Contrasting Transcriptome Dynamics in Response to Extracellular Self vs. Nonself-DNA

The transcriptome analysis of plants treated with self-DNA showed a limited number of differentially expressed genes compared to the exposure to nonself-DNA, although remarkable GO enrichments could be revealed.

After one hour, a primary response to self-DNA sensing is the enrichment of upregulated genes involved in the chloroplast class (structure and photosynthesis groups) but with the lack of differential expression of nuclear genes related to chloroplast activity (genome and light harvesting groups). Significant enrichments are evident also in NAD/NADP related processes, proton and electron transport and ATP related processes, all due to upregulated DEGs, with the ATP related processes group evident only in self. Interestingly, there is no evidence of enrichment in the GOs related to mitochondrion. Nevertheless, among the 146 mitochondrial related genes, 7 are expressed in self and they are all upregulated, e.g., the mitochondrial gene RPS3 (ATMG00090) encoding a ribosomal protein related to pathogen resistance [64], and the nuclear gene AOX1d (AT1G32350) [32], as also confirmed by the QRT-PCR, which is typically upregulated in response to stress [65]. In particular, AOX1d contributes to the recovery from the inhibition of Complex III that is involved in the mitochondrial electron transport chain, thus indicating a block of the respiratory chain typical of the self-DNA treatment. The upregulation of AOX1D is coherent with the presence of nitric oxide (NO), as reflected by the upregulation of both NIA2 (Nitrate reductase: AT1G77760) and NIA1 (AT2G15620). The latter being related to the downregulation of AT2G28160 and AT3G25190 that are associated with the inhibition of ethylene production and generally associated with NO activation. NO is an alternative ROS product, determined by a drop of the oxygen concentration, also activating 2 oxoglutarate [66] and determining the inhibition of aconitase [67], which ends up with the upregulation of AOX1D. Noticeable, although the higher expression of ERF2 at the first hour, the down regulation of specific genes involved in ethylene signal transduction (AT4G34410, AT5G52020) and ethylene responsive transcription factors (AT1G74930, AT1G28370) is remarkable at 8 h in self treatments.

In addition, the hyper-activation of the chloroplast genome activity, in absence of a similar upregulation of chloroplast proteins encoded by the nuclear genome, reveals further specific peculiarities due to self-DNA exposure. The upregulation of chloroplast encoded genes, that does not meet a corresponding gene expression in nuclear encoded genes, could cause the overproduction of chloroplast related ROS, also caused by the lack of overexpression of genes like ascorbate peroxidases, namely the APX1 gene (AT1G07890). Ascorbate peroxidases acts as scavengers of H_2_O_2_ in the chloroplast, moreover suppressing the expression of H_2_O_2_-responsive genes under photo-oxidative stress [68]. This is also known to be accompanied by a downregulation of HSFA2 [41], which could explain the evidence here reported of an unexpected pattern of downregulated heat shock related proteins, as revealed for self-DNA exposure at 8 h.

However, further investigations should clarify the discrepancy here highlighted by the QRT-PCR that also shows a downregulation of the HSFA2 at the 8-h port treatment also in the nonself treatment that is not accompanied by the downregulation of heat shock proteins. 

Interestingly, the upregulation of chloroplast related genes (involving proton and electron transport, ATP-related processes and structural components of chloroplast) observed at 1 h post exposure in self-DNA treatment, completely turned off after 8 h.

The enhanced ROS production starting at the first hpt in self, revealed by the specific signatures that witness these events and also reported as production of H_2_O_2_ in similar experiments of exposure to self-DNA [27], does not exclude the formation of singlet oxygen O^2-^, because of the activation of superoxide dismutases in self. The O_2_ drop down—maybe contributing to NO formation—is also accompanied by the downregulation of ethylene responsive transcription factors revealed at 8 hpt to self-DNA. 

At 16 h, genes encoded by the chloroplast genome showed again upregulation in self-DNA treatments, indicating a recursive effect and a persisting stressing stimulus. It will be of interest, in future efforts, to monitor the hormonal as well as NO and H_2_O_2_ waves possibly accompanying the process. Moreover, photoinhibition should also occur, deteriorating the chloroplast machinery for longer exposure.

Considering the classes of genes specifically related to responses to stresses (biotic and abiotic stress, and systemic resistance) common DEGs are detected for both treatments. Nevertheless, the number of specific genes highlight initial milder differences between the two responses (Figure 4), while discrepancies become more evident in the two later stages. Indeed, self and nonself treatments, respectively, show along the three stages after treatment: 15-8-3 versus 22-44-67 DEGs in the biotic stress; 35-58-22 versus 68-199-237 for abiotic stress; 1-2-1 versus 4-8-11 in the systemic resistance.

In particular, genes related to heat, wounding and chitin response were downregulated, while responses to oxidative stress, toxic substances and ions were upregulated in self, involving genes encoding detoxification and anti-oxidation protective enzymes [69,70,71,72]. Such results clearly indicate that self-DNA triggered a response to oxidative stress and detoxification, while downregulating typical stress responsive genes, like HSPs, as it resulted evident at 8 h. 

Downregulated genes in self at the first hpt include PAD4 (AT3G52430: phytoalexin deficient 4), as also confirmed by QRT-PCR, that usually, when upregulated, mediates TIR-NB-LRR signalling involved in the pathogen resistance response. The downregulation of PAD4, indeed, may be associated with the inhibition of TIR-NB-LRR signalling involved in the resistance responses mediated by TIR containing R proteins [43]. On the other hand, the upregulation of the systemic resistance and biotic stress responses is evident after exposure to nonself-DNA. Indeed, PAD4 (AT3G52430: phytoalexin deficient 4) is upregulated in nonself. Precisely, the self-specific downregulation at the first hour and the nonself-specific upregulation at 16 h, as also confirmed by QRT-PCR, also accompanied by the upregulation of genes involved in the hypersensitive response (AT3G52430, AT2G38470, AT1G91560, AT3G45640, AT5G07390, AT1G01480, AT4G11280 and of several TIR-NBS-LRR proteins), indicates the triggering of the related processes as a nonself-specific phenomenon. Moreover, an upregulation of genes related to systemic acquired resistance is also detected in this stage in nonself (AT3G45640, AT2G38470, AT1G19250, AT2G13810, AT3G56400, AT5G26920 and AT1G73810), consistent with the hypothesis that nonself-DNA acts as a PAMP triggering plant immune response [26], although we could not detect differential expression for enhanced disease susceptibility 1 (EDS1) and the senescence-associated gene 101 (SAG101) complex, that usually are also involved with PAD4 in triggering the two processes [43]. Overall, on one hand these findings suggest that the effects of nonself-DNA recall a PAMP-like response, as it is evolving towards a systemic acquired resistance, that is not revealed from our results from self-DNA treatments, the latter being possibly more related to a DAMP like response [23].

Additionally, the GO enrichment analysis indicates an evident upregulation of most genes involved in local or systemic response in nonself treatments, while self-DNA treatments highlight the absence of a hypersensitive response which is also accompanied by early upregulation of genes related to both ABA and jasmonic acid only in the first hpt. 

Considering other hormone related responses, the results of GO enrichment analysis indicated a consistent upregulation of genes related to cytokinin and brassinosteroids and a downregulation of gibberellins in treatments with self-DNA. Differently, in the case of nonself-DNA, hormonal response trends revealed by the GO analysis were limited to an upregulation of genes related to ABA and salicylic acid. The upregulation of most of the genes belonging to the cytokinin oxidase/dehydrogenase (CKX) family in the self-DNA treatment indicates cytokinins breakdown and suggests that at 16 h cytokinin-mediated processes are negatively affected, possibly involving, among others, cell cycle regulation, cell proliferation and shoot and root development [73]. 

Self-DNA treated plants also showed downregulation of gibberellins transport. Interestingly, two loci (AT4G25010, AT5G50800), SWEET13 and SWEET14, are members of the SWEET family, known for including a major class of sugar membrane transporters in plants [74,75]. However, a recent study clarified an additional, interesting function of these two carriers, which can transport gibberellins at intra- and inter-cellular levels, thus possibly affecting plant development and growth [76]. In the same study, the highest levels of the proteins AtSWEET13 and AtSWEET14 were found in roots of 1-week-old seedlings. Our finding of a downregulation exclusive of the self-DNA treatment after 16 hpt may be related to the growth inhibition observed at a later stage in our analyses (Figure 6) that confirmed what previously highlighted [1]. 

### 3.2. Hypotheses on the Mechanisms of Self-DNA Inhibition in Plants

The discovery of plant growth inhibition by self-DNA [1] could be the result of a mechanism resembling “processes of interference based on sequence-specific recognition of small-sized nucleotide molecules” [1], thus explaining the specificity and determining inhibition of the cell functionality [7]. A further hypothesis was suggested to explain the dosage-dependent growth-inhibition by self-DNA as the phenotypic consequence of a costly immune response [8,23]. However, it has been already underlined that “the molecular mechanism underlying growth inhibition by eDNA… is uncharacterized” [9].

Self-DNA fragments that appear in the extracellular space have been suggested to act as DAMPs: i.e., endogenous signals of danger that indicate the disruption of cell integrity [27,77]. Mechanical damage, feeding by chewing herbivores (including hydrolases in their saliva) and even infection by necrotrophic pathogens cause the disruption of cells, and the subsequent release of ATP, small signalling peptides (AtPeps), or cell wall fragments, and all these DAMPs thus activating the plant response [49,51,78,79,80]. This signalling cascade comprises membrane depolarization, Ca^2+^ fluxes, ROS production and MAPK activation and the subsequent induction of a JA dependent broad spectrum immunity against chewing herbivores and necrotrophic pathogens [81]. The JA-dependent immune response causes dosage-dependent metabolic costs which, at the phenotypic level, may become apparent as stunted growth or a transient growth arrest [82,83,84]. Under this scenario, the general assumption is that the immunogenic properties of self-DNA and other DAMPs correlate with their dosage-dependent inhibitory effects on growth. Indeed, DAMPs trigger ROS, ethylene production and JA signalling in *A. thaliana* [85,86,87,88,89,90] with more than half of the DAMP-induced genes shared with JA-signalling [87,88,90]. Mechanical wounding of common bean (*Phaseolus vulgaris*) leaves resulted in enhanced levels of eATP, which triggered the production of ROS and the activation of catalase and polyphenol oxidase [91]. Correspondingly, fragmented self-DNA triggered membrane depolarization in maize (*Zea mays*) and lima bean (*Phaseolus lunatus*) [5], ROS production, MAPK activation and JA increase in common bean [27], and the expression of superoxide dismutase, catalase, and phenylalanine ammonia lyase in lettuce (*Lactuca sativa*) [25]. Intriguingly, herbivores might even secrete DNases to suppress the self-DNA-triggered plant immune response [92]. In accordance to the “cost” hypothesis, eATP and AtPeps strongly inhibited *A. thaliana* seedling growth in several studies, and this was suggested to be a direct and causal relation of the immunogenic and growth inhibiting effects of eATP or AtPeps [89,93,94]. Additionally, for self-DNA, growth inhibition correlates with immune responses in common bean [27] and *Lactuca sativa* [25].

However, consistent with our results on the upregulation of the response to biotic stress prevailing in the nonself treatments, together with the triggering of the hypersensitive response in the later stages of this treatment, nonself-DNA would be sensed as a PAMP and not a DAMP. PAMPs also trigger a growth arrest/inhibition that can be associated with their immunogenic effects [89,95,96,97]. However, growth reduction is not generally reported for nonself-DNA treatments, although few examples exist: nonself-DNA from bacteria triggered ROS and callose deposition in *A. thaliana* seedlings and strongly inhibited their growth, and nonself-DNA from herring triggered the same response, although to a lower degree [95]. In some cases, growth inhibition was also observed by heterospecific DNA from phylogenetically related plant species [1,25,27]. However, self-DNA always caused a stronger inhibitory effect than nonself, with the taxonomic distance between the exposed species and the species used as source of the DNA playing a significant role on the extent of the inhibition, thus giving origin to consider as self-DNA treatments, also those involving “homologous” DNA. In particular, exDNA from *A. thaliana* inhibited the growth of *Lepidium sativum* seedlings and vice versa, but DNA from *A. thaliana* did not inhibit *Acanthus mollis* growth [1]. Interestingly, *A. thaliana* and *Lepidium* belong to the same order (Brassicaceae), whereas the Acanthaceae belongs to a different order, the Lamiales. Similarly, DNA from *Capsicum chinense* inhibited *Lactuca sativa* (both Asterales), whereas DNA from *Acaciella angustissma* (Fabales) did not [25], and DNA from lima bean inhibited common bean growth whereas DNA from *Acacia farnesiana* did not [27]. 

ExDNA from phylogenetically unrelated species was even reported to promote growth, being used as a phosphorous source [22]. The easiest explanation for these observations would be that the growth-inhibitory effect of self-DNA could be linked to a mechanism recognizing self as well as “homologous” DNA, i.e., DNA from phylogenetically related species, although the latter is recognized to a lower extent [1,2,7,8,23,98]. Although generalizations may be limiting, it would have been more intuitive to expect a more costly response to foreign DNA, rather than to self or to closely related DNA. This could be also in agreement with the stronger molecular response, in terms of transcriptome changes, here revealed in nonself rather than in self treatments. However, this evidence appears in contrast with the hypothesis of the growth inhibition in self treatments as a consequence of the metabolic cost of the immunity response. As an alternative hypothesis, Mazzoleni et al., 2015 [1] and later Cartenì et al., 2016 [7] suggested a different explanation based on a more direct effect, i.e., the possible “interference” of extracellular self- or “similar” DNA (e.g.: homologous, i.e., from phylogenetically related species or even similar, i.e., with convergent structure similarity, although not phylogenetically related) causing inhibition of the whole cell functionality, mediated by sequence-specific recognition of small-sized nucleotide molecules [99], which could hamper cell and gene expression functionality [100] or affect genome stability [101], inhibiting the growth. This could explain the self-DNA growth inhibition as a widely conserved property of living beings, and therefore justify its observation over a very wide range of organisms spanning from prokaryotes to metazoan [2]. On the other hand, it would be difficult to assume that specialized molecular machineries across several kingdoms, including immunity response at cell level, would have evolved and remained conserved to constantly produce similar but highly specific growth inhibition effects in all species (e.g., metabolic cost of immunity response). Consequently, the inhibitory effect of self-DNA has to be explained by a more general and basic recognition mechanisms, inhibiting cellular activities, leading to the production of ROS, causing cell or DNA damaging effects [102], determining cell cycle arrest and growth inhibition at the macroscopic level. In particular, it would be interesting to investigate how these mechanisms are associated with the more general frame of epigenetic responses to stress, including chromatin organization and its effect on genome stability, and related methylation profiles [103].

### 3.3. EDAP: Extracellular DNA Associated Pathways

We here demonstrate that the cell is able to sense exDNA distinguishing between self and nonself DNA, in plants. This is revealed by our observations from fluorescence microphotography, that indicates different patterns of exDNA localisation, with nonself-DNA (non-similar or phylogenetically distant) entering root tissues and cells, while self-DNA (conspecific and/or similar or “homologous”) remaining outside. Furthermore, the transcriptome analysis reveals that specific and different molecular pathways are triggered by the early response to self- and nonself-DNA, respectively. We propose to define these pathways as extracellular DNA associated pathways, suggesting the new acronym EDAP. This acronym is useful to depict the differential response to self and nonself DNA exposure, since no specific pathway was described before that could explain the difference between the two categories of molecules (self and nonself DNA, respectively). Specifically, our findings show, on one hand, in the case of nonself-DNA a remarkable differential gene expression, involving both biotic and abiotic stress related genes, accompanied by the mounting of a hypersensitive response, putatively triggering a systemic acquired resistance. On the other hand, a minor differential expression is evident in the self-DNA response that is, however, remarkably associated with oxidative stress, and the activation of the chloroplast genes, with the down-regulation of stress responsive genes. The self-recognition is known to trigger an early intracellular Ca^2+^ spike signal and cell membrane depolarization, as observed 30 min after exposure to self-DNA [5]. This may be accompanied by the downregulation in signal transduction related genes, as here revealed by the transcriptome analysis. Among these, the CBL-interacting protein kinases, which are known to be active in the Ca^2+^ dependent signalling cascades [104] once bound to Calcineurin B-Like proteins (CBL), also regulate the response to oxygen deficiency and osmotic and salt stress [105,106]. This may suggest decreasing intracellular dynamics, possibly affecting the crosstalk between the chloroplast and the nuclear genome activities, after self-DNA exposure, that can be coupled with the activation of cation cotransporters, such as the vacuolar H^+^/Ca^2+^ antiporter [107]. 

**Figure 7:**
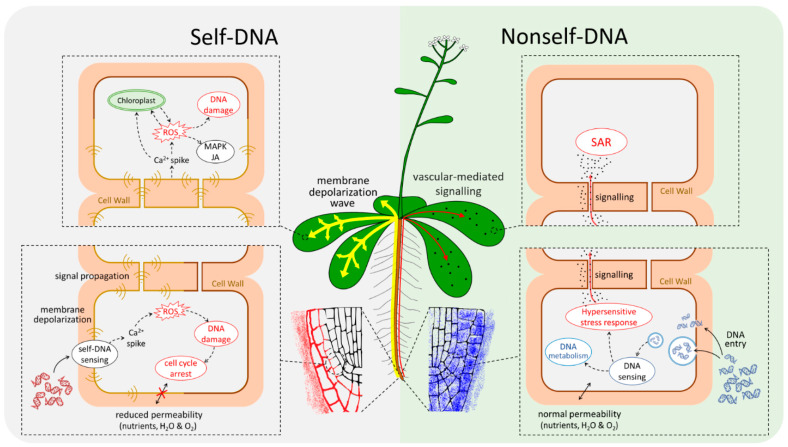
Model of Extracellular DNA Associated Pathways (EDAP) in plants. The exposure to either self- or nonself-DNA produces differential cellular responses. The self-DNA treatment triggers an electric response, starting with a sensing at membrane level, with calcium spikes followed by a reduced permeability of the roots, and a cascade of events involving the chloroplasts and inducing ROS production. On the other hand, nonself-DNA enters the cells where it is metabolized activating a cascade of events inducing a hypersensitive response.

After the initial sensing, that can be in principle ascribed to the category of DAMP sensing, since self-DNA is a damage associated molecule, the signal can be rapidly propagated throughout the plant [108,109,110,111] and, once reaching photosynthetic organs, it can also induce the activation of the chloroplast genome (Figure 7), as clearly depicted by the transcriptome analysis and described here for the first time. The lack of the up-regulation of genes acting as chloroplast ROS scavenging, presumably due to the inhibition of the chloroplast-nuclear cross-talk here hypothesized, induces a positive feedback on ROS production which is associated with the MAPK activation and JA production that was reported in other experiments of exposure to self-DNA [27]. The abovementioned inhibition is also reported to induce ROS production. This is linked to O_2_ drop down that may contribute to the activation of the NO pathway determining the downregulation of ethylene response after exposure to self-DNA. We hypothesize that the inhibition of the intracellular dynamics and crosstalk can be a typical initial cell response after the sensing of external self damage patterns, with the length of the exposure to the stressor agent strongly affecting cell fate and the extent of the damage to the whole organism. 

To the best of our knowledge, self or nonself specific DNA receptors have not yet been described for plants so far [9,26,77]. However, among the putative eDNA/eRNA receptors described as DAMPs in *A. thaliana* [9,49,50,51,52,53,54,55,56,57,58,59,60,61,62,63,64], only three resulted upregulated at the first hpt in self, among which AT1G64160, that is a DEG also in the nonself treatment, in contrast with the 11 upregulated specific DEGS resulting from the nonself treatment.

DNA fragments may enter the cell, directly interfering with gene expression, as previously shown, e.g., for triplex-forming oligonucleotides (e.g., [112]) or even interacting at cytoplasmic level with redundant metabolic DNA [113]. However, our fluorescence microphotography experiments did not provide support to such hypothesis in the very early stage of the response to self. Indeed, the results here presented pave the way to consider that the sensing should occur in the extracellular environment, possibly on the extracellular surface of the plasma membrane or on its immediate surroundings, explaining the reasons of the accumulation of extracellular self-DNA on cell surfaces in contrast with the entrance and the active endocytosis stimulated by the nonself-DNA, that can also clarify the reasons of different molecular mechanisms associated with the different molecular responses, although further investigation should address possible explanations. On the basis of such logical reasoning, we speculate that nucleic acids themselves, known to be present in the extracellular environment could be involved in the self/nonself discrimination in plants and presumably in all living beings [21]. However, further validation on the location and the nature of the involved receptors or sensing mechanism still deserves further investigations to clarify if the sensing mechanism of exDNA, and specifically of self-DNA, could occur outside cells, possibly inhibiting successive processes like endocytosis or intracellular cross-talks to favour recovery from “damages” highlighted by the presence of “unexpected” self exDNA.

Our study clearly demonstrated that exposure to self-DNA produces a dramatic change of the biophysical state, directly determining the inhibitory effect, while no evidence of a costly activation of an immune response could be detected over our observation time frame. 

Our analysis on possible DAMP and PAMP receptors or expression pathways, did not highlight clear trends in the context of known responses, but a more complex picture emerged for what concerns the response to extracellular DNA, clearly depicting the different response to self or nonself-DNA. This preliminary evidence surely needs more investigations and further assessments to better characterize what we here propose to describe as Extracellular DNA-Associated Pathways, introducing the acronym EDAP, that need to explain overlap and discrepancies in the sensing of self and nonself-DNA, highlighting novel perspectives on this subject.

## 4. Materials and Methods

### 4.1. DNA Extraction and Preparation

DNA was extracted from both *A. thaliana* young leaves and *Clupea harengus* (common herring) abdominal muscles with the same procedure. One gram of tissue was ground in liquid nitrogen and then mixed in CTAB buffer (0.1 M Tris-HCl pH 7.5, 0.7 M NaCl, 0.01 M EDTA, 1% CTAB). Samples were incubated 45 min at 65 °C and mixed periodically. Chloroform/Isoamylalcohol (24/1) was added to equal volume of sample mixed and centrifuged 20 min at 14,000× *g*. The upper phase was collected and transferred to a new tube for precipitation with 1 volume of Iso-propanol at −20 °C for 1 or more hours. Samples were centrifuged at max speed, at 4 °C for 20 min. The upper phase was discarded, and pelleted DNA was washed with cold 70% ethanol in a centrifuge at max speed for 20 min. Ethanol was discarded and DNA was air dried and resuspended in 200 µL of sterile water. RNAse A (Thermo fisher, Third Avenue Waltham, MA, USA) was added at a final concentration of 0.25 mg/mL and incubated at 37 °C for 1 h. RNAse A was inactivated at 70 °C for 20 min. 

The DNA was extracted with the same protocol from different tissue types of each species to randomize and overcome biases due to the methodology employed. Two independent DNA extractions were performed per sample (technical replicate). The DNA samples used as treatments were firstly checked by nanodrop standard quality parameters (260/280 and 260/230 ratio above 1.8) to evaluate DNA purity. The DNA quantity was evaluated using a QUBIT (Thermo Fisher, Third Avenue Waltham, MA, USA) fluorimeter, and its integrity was evaluated by electrophoresis in 1% agarose gel. The DNA was sheared using a Bioruptor Plus (Diagenode, Seraing (Ougrée) Belgium, EU), for 12 min at high power, setting 60 s ON and 30 s OFF in order to reach an average size ranging from 200 to 700 bp. Such size was selected following previous evidence that this range was that found in decomposing litter and the most effective to produce inhibitory effects in vitro conditions [1].

### 4.2. Plant Materials and Treatments

*A. thaliana* (L.) Heynh. Col-0 (186AV) seeds were obtained from the “Centre de Ressources Biologiques” at the “Institut Jean Pierre Bourgin”, Versailles, France (http://dbsgap.versailles.inra.fr/vnat/). Seeds were treated in 70% ethanol for 30 s, then transferred in a sodium hypochlorite (1:10 of commercial concentration) and 0.05% tween 20 solution for 10 min with occasional vortexing, washed with sterile milli-Q water 4 to 5 times, dried and resuspended in a sterile agar solution (0.2%). Sterilized seeds were vernalized for three days at 4 °C and prepared for sowing.

For transcriptome analyses, Petri dishes with a layer of thin Whatman paper were prepared in sterile conditions for each sample. The dishes were wetted with pure sterile water and an adequate number of Col-0 sterilized seeds were sown. The plates were put in the dark for 48 h in a growth chamber with 50% controlled humidity. After two days the plates were kept in 16h/8h light/dark cycles in controlled humidity conditions. If needed, sterile water was added to the plates from time to time, to all samples. 

The experimental design for both transcriptome (Figure S2) and confocal analyses included two replicates of the following treatments: (1) control: with sterile distilled water; (2) self-DNA: with *A. thaliana* DNA; (3) nonself-DNA: with *Clupea harengus* DNA. Both DNA treatments were performed using a concentration of 200 ng/µL (as in [1]).

Before both transcriptome and confocal experiments, plants were grown for five days till the two true leaves stage. 

For confocal analyses, *A. thaliana* plants (Col-0) were grown vertically on half strength MS basal medium. Five-days-old seedlings were placed on slides. They were treated with self and nonself-DNA and observed after 1 h.

For transcriptome analysis (summary in Figure S1), plants were grown until the appearance of the first true leaves. At this stage, the filter papers were imbibed with the control solution, 1.2 mL of sterile distilled water, or the same volume of 200 ng/µL of sonicated DNA (self or non self). Control and treated plates were harvested at 1, 8 and 16 h (two biological replicates per treatment), then immediately frozen with liquid nitrogen. Additional plants that were not harvested for RNA extraction were maintained for 15 days for final observations on longer terms phenotypic effects. 

### 4.3. Transcriptome and Bioinformatics Analyses

Total RNA extraction from harvested plant material was performed using RNeasy micro kit from QIAGEN (Cat No./ID: 74004) following the standard extraction protocol and sent to a service provider for the RNA-seq analysis on Illumina Hiseq2500, by single read sequencing 1 × 15M.

Raw reads per sample were cleaned from adaptors and low-quality bases using the Trim Galore package (http://www.bioinformatics.babraham.ac.uk/projects/trim_galore/, access time: 3 September 2014), applying the default settings for single read sequencing. The cleaned reads were then mapped to the Arabidopsis nuclear and cytoplasmic genomes (version TAIR 10) using the STAR aligner software (version 2.4.2a) [114] allowing a maximum number of 10 mismatches.

Detailed results from the pre-processing are reported in Table S1. The mapped reads were counted per exon by featureCounts (version 1.4.6-p5) [115], using the strand specific count (“-s 2” option) and allowing the counting of read on overlapping features for each feature (“-O” option).

Assessment of replicates correlation in terms of RPKMs was performed by Pearson correlation and is reported as the correlation matrix and the associated dendrogram (Figure S3a,b). The principal component analysis of the same data per treatment per time is also shown (Figure S3c). DEGs call, comparing DNA treatments at each stage with the respective control, were made performing three different statistical approaches (FDR < 0.05): (i) DESeq2 [116]; (ii) edgeR and (iii) edgeR GLM The union of the three approaches was considered for subsequent analyses.

A K-means cluster analysis on log2 fold changes of all genes that resulted DEGs in at least one treatment/stage, was performed using MeV [115], by the Pearson Correlation as distance metric, setting at 15 the number of requested clusters according to the Figure of Merit (Figure S4). DEGs and samples were also submitted to PCA ordination, then plotting vector loadings for treatment combinations (timing and type of exposure to DNA) and factorial scores of cluster centroids in the multivariate space defined by the first three ordination axis. 

GO enrichment analyses on DEGs filtered by |log_2_(FC)|≥ 1 were performed using the GOseq package [116] (FDR ≤ 0.05), and the reference GO annotation for Arabidopsis that was downloaded from Ensembl Plants (http://plants.ensembl.org/index.html, access time: 7 September 2018). 

The GO enrichment analysis was also performed on the clusters obtained from the statistically significant DEGs, too. In order to better represent the prominent GO terms per cluster, selected keywords from the most enriched GOs were used to build word clouds (https://www.wordclouds.com/, access time: 11 November 2018), where the size of the represented words is directly proportional to the number of times they appear in the input data.

Lists of genes annotated with the most enriched GOs, showing different expression patterns between self-DNA and nonself-DNA treatments at each observation stage (i.e., 1, 8 and 16 h) were collected in order to quantitatively assess between-treatment differences and analyse their contribution in detail at single-gene level.

### 4.4. Confocal Microscopy Experiment 

The confocal analyses were performed independently in two different institutes by using three different confocal microscopes (Zeiss LSM-700, Leica TCS and Zeiss LSM-780), in order to confirm and verify the reproducibility of the results. 

The analyses were conducted treating roots by a DNA concentration of 100ng/µL DNA that included labelled DNA by Cy3 dye for an incubation period of 1 h. Seedlings were then transferred to a new slide and treated with 2 µM FM4-64 for 5 min. Before the confocal observations, seedlings were subjected to two sequential washing steps using tap water. Then, they were transferred to the slide for the confocal analyses. As a negative control, Arabidopsis seedlings were incubated for 1 h with the same amount of Cy3 (without DNA), then exposed to the same treatment with FM4-64. The images were analysed and edited by using the ImageJ free software.

### 4.5. QRT-PCR Validation

Seven target genes were selected considering their behaviour as DEGs in at least one stage/treatment and checked for duplication in the TAIR database (Arabidopsis.org). Using the RealTime qPCR online tool from IDT (https://eu.idtdna.com/scitools/Applications/RealTimePCR/, access time: 3 March 2021). One pair of primers was designed for amplification of the selected genes targeting the exon-exon junction, when possible, to exclude intronic regions arising from genomic DNA or not mature mRNAs. The 1st strand cDNA was synthesized with superscript III (ThermoScientific) starting from 600 ng of the same RNA samples used for the RNA-seq analysis and using manufacturer conditions. Each primer pair was verified in PCRs using first-strand cDNA as template. PCR conditions were as follows: denaturation at 94 °C for 4 min, cycling at 94 °C for 30 s, annealing at varying temperatures for 30 s, and extension at 72.0 °C for 30 s. Annealing temperatures ranged from 50 °C to 68 °C. Reaction products sizes and integrity were visualized by 1.2% agarose gel electrophoresis.

qPCR was performed in 20-µL reaction volumes containing 10 µL Power SYBR™ Green PCR Master Mix (Thermo-Fisher Scientific; catalog no. 4367659, Third Avenue Waltham, MA, USA), 500 nM forward and reverse primers and 1:10 dilution of 600 ng stock sample cDNA. 

For each gene, we considered two biological replicates with two technical replicates per biological replicate. qPCR amplifications were performed on separate plates, where each plate contained primer pairs for the housekeeping gene GAPDH (AT1G13440.1, Jin et al., 2018). qPCR expression values were calculated for each gene as the difference between the quantification cycle of the gene and the reference gene, averaged over technical replicates (Table S7). Concordance between RNAseq and RT-qPCR results was assessed by testing the statistical significance of linear regression of the log2(FC) versus the log2(ΔΔCt) results, for those transcripts for which both metrices were available (n = 42, 7 transcripts × 2 DNA treatments × 3 time points). For each metric, the mean of replicated values was used for each combination of transcript, DNA treatment and observation time. Evident outliers were removed before fitting the regression model (Table S7).

## 5. Conclusions

In this work, we confirmed that the early molecular response to self-DNA is observable before the phenotypic effects of inhibition on root growth become evident, and that it can be clearly distinguished from the one to nonself-DNA. In the proposed model, self-DNA sensing at root level induces a membrane depolarization wave that rapidly propagates throughout the plant, leading to reduction of cell permeability and eventually to DNA damage and cell cycle arrest. Differently, nonself-DNA enters the cells eliciting a remarkable differential gene expression. This is associated with the activation of the hypersensitive response, possibly evolving into a systemic acquired resistance.

Mazzoleni and collaborators [1] reported that when plants are exposed to decomposing litter of phylogenetically similar species the inhibitory effect is still found, even if at a lower level. These observations, together with the need of deeper view on the mechanism through which the self- and nonself-DNA act, raise the interest for further investigations, both at transcriptomic and microscopic levels, of the A. thaliana response after the exposure to nonself-DNA at different degree of phylogenetic distance. Our results provide additional hints to the scenario concerning exDNA functions and its effects on plants, presumably justifying the same effects also in other species. They pave the way to additional investigations to demonstrate the mechanisms of exDNA sensing at extracellular level, and the specific recognition of self and nonself DNA in relation to the phylogenetic distance between the stimulating molecules and the structure features of the exposed organism genome. Finally, considering that self-DNA inhibition was demonstrated in many species across different kingdoms, further research should address the characterization of the EDAP in different model organisms, including prokaryotes and other eukaryotes. 

## Data Availability

Original datasets presented in this study can be found in the Sequence Read Archive (SRA), with the following accession identifier: BioProject ID: PRJNA707115.

## Supplementary Materials

The following are available online at https://www.mdpi.com/article/10.3390/plants10081744/s1, Figure S1: Correlation analysis between log2FC RNA-seq and log2 ΔΔCt of QRT-PCR. The correlation was done using Person correlation, the R2 is also reported, Figure S2: Schematic representation of the experimental design, Figure S3: Correlation analysis (A) of gene expression (in RPKM) in all replicates (Control (C01, C08, C16), Self (S01, S08, S16), and Nonself (N01, N08, N16) indicate each stage per treatment. The associate cluster dendrogram (B) and the principal component analysis (PCA) (C) are also shown, Figure S4: Figure of Merit. The most suitable number of clusters expected per fold change of genes resulting in be DEGs in at least one stage per the two treatments, in a K-means clustering, is indicated by the red vertical bar, corresponding to 15 on the abscissa axis, Table S1: Summary of the RNA-seq pre-processing results showing, for each replicate, the total number of raw reads and the percentage of reads discarded after the cleaning steps and uniquely mapped on the Arabidopsis thaliana genome, Table S2: (a) Number of total genes and number of DEGs with assigned GO per treatment. (b) Number of common and specific DEGs in self-DNA and nonself-DNA treatments at 1, 8 and 16 h post treatment (hpt), Table S3: (a) List of statistically significant differentially expressed genes (DEGs) in self and nonself-DNA treatments and average RPKM values. Log2FC are shown for all DEGs. DEGs with a Log2FC ≥|1| are indicated in colour cells: in red if upregulated and in blue if downregulated. (b) List of statistically significant differentially expressed genes (DEGs) in self and nonself-DNA treatments and average RPKM values. Log2FC are shown for all DEGs. DEGs with a Log2FC ≥|1| are indicated in colour cells: in red if upregulated and in blue if downregulated, Table S4: Enriched Gene Ontologies (GOs) in self and nonself-DNA treatments organized in classes and groups. In red (UP) or in blue (DOWN) the GOs enriched by up or downregulate filtered DEGs (expression pattern) are shown, Table S5: Results of the cluster analysis based on fold changes in all stages of genes that are differentially expressed (DEGs) in at least one of the six treatments (2 DNA sources × 3 observation times). From red (high) to blue (low) the level of fold change values is shown, Table S6: (a) Number of filtered DEGs per class of GOs in self and nonself-DNA treatments at 1, 8 and 16 h post treatment (hpt). (b) Number of filtered DEGs per hormones in self and nonself-DNA treatments at 1, 8 and 16 h post treatment (hpt), Table S7: List of Genes that were significantly differentially expressed (DEGs) in at least one treatment/stage selected for validation by RT-qPCR, Table S8. (a) List of DAMPs and PAMPs in A. thaliana. (b) A summary of DEGs number per DAMP (525 genes in total) or PAMP (36 genes in total) classes of responsive genes.
