# Supplementary material for: Arabidopsis thaliana Response to Extracellular DNA: Self Versus Nonself Exposure
Source: Plants (Basel). 2021 Aug 23;10(8):1744. doi: 10.3390/plants10081744 (PMC8400022; doi:10.3390/plants10081744)
Supplement: Supplementary file 1 [file plants-10-01744-s001.zip › Supplementary Figure.pdf]

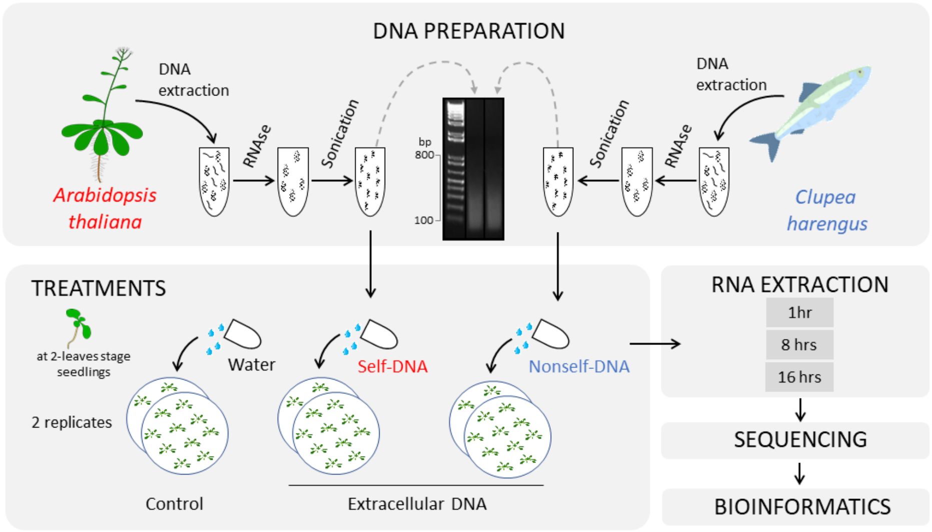

Figure S1: Correlation analysis between log2FC RNA-seq and log2 ΔΔCt of QRT-PCR. The correlation was done using Person correlation, the R2 is also reported

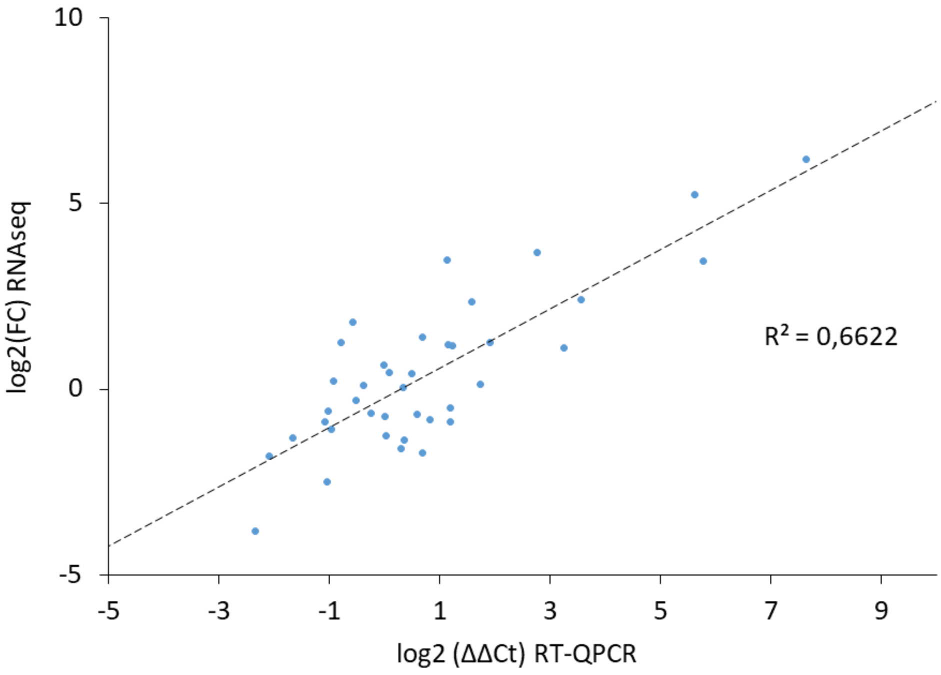

Figure S2: Schematic representation of the experimental design

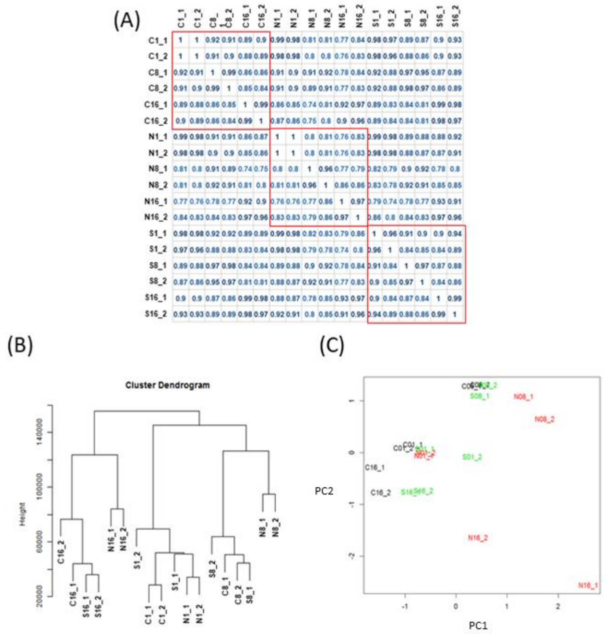

Figure S3: Correlation analysis (A) of gene expression (in RPKM) in all replicates (Control (C01, C08, C16), Self (S01, S08, S16), and Nonself (N01, N08, N16) indicate each stage per treatment. The associate cluster dendrogram (B) and the principal component analysis (PCA) (C) are also shown

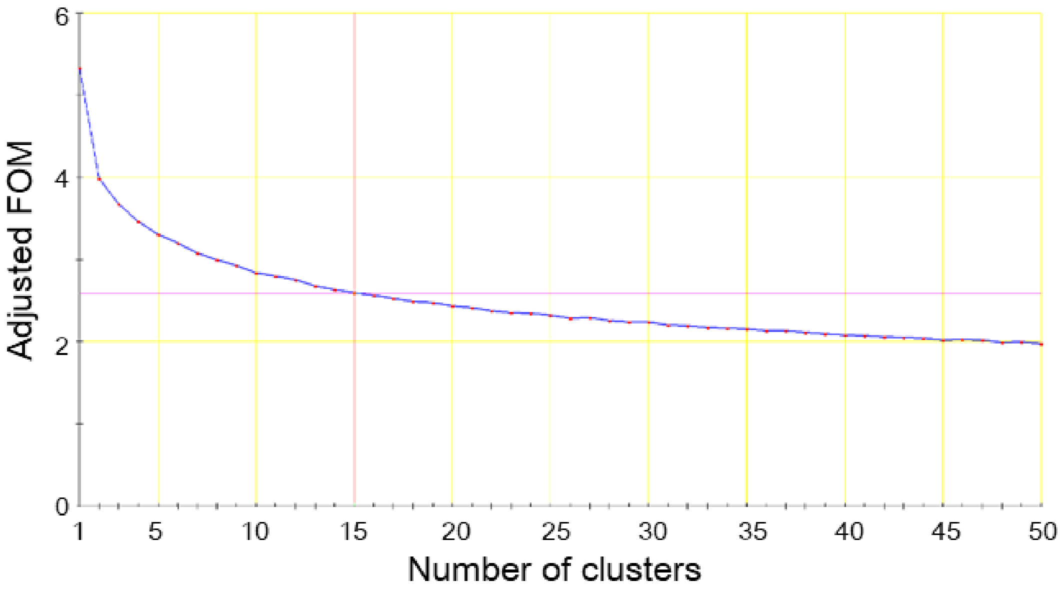

Figure S4: Figure of Merit. The most suitable number of clusters expected per fold change of genes resulting in be DEGs in at least one stage per the two treatments, in a K-means clustering, is indicated by the red vertical bar, corresponding to 15 on the abscissa axis
